# Oxygenation and Spatial Heterogeneity Shape Radiotherapy Protocol Ranking Through Phenotypic Adaptation

**DOI:** 10.1007/s11538-026-01758-5

**Published:** 2026-10-03

**Authors:** Francesco Albanese, Giulia Chiari, Marcello Edoardo Delitala

**Affiliations:** 1 Department of Mathematical Sciences “G. L. Lagrange”, Politecnico di Torino, Torino, Italy; 2 Basque Center for Applied Mathematics (BCAM), Bilbao, Spain; 3 Mathematical Institute, University of Oxford, Oxford, UK

**Keywords:** Radiotherapy fractionation, Phenotypic and spatial heterogeneity, Radioresistance, Tumor hypoxia, Evolutionary dynamics, Mathematical oncology

## Abstract

Tumor response to radiotherapy is strongly influenced by oxygen availability and phenotypic heterogeneity, yet their combined impact on the relative performance of fractionation schedules remains unclear. Here, we develop a mathematical model that integrates spatial oxygen dynamics with continuous phenotypic adaptation to hypoxia and radiation, and use it to systematically compare radiotherapy protocols under a common normal-tissue toxicity constraint. Under spatially uniform oxygenation, we find that alternative fractionation schedules provide little improvement over standard-of-care protocols in normoxic conditions. Under moderate hypoxia, however, a distinct class of protracted schedules with longer inter-fraction intervals substantially increases time-to-progression, in some cases by up to twofold. This regime-dependent benefit is consistent with a shift in the balance between reoxygenation and selection for resistant phenotypes. When oxygen delivery is spatially heterogeneous, treatment outcomes depend strongly on the geometric organization of oxygen sources. Even with identical total oxygen supply, different spatial configurations lead to large variability in time-to-progression and can alter the relative ranking of radiotherapy protocols. These results show that radiotherapy effectiveness is not an intrinsic property of a treatment schedule alone, but emerges from its interaction with tumor microenvironmental structure and evolutionary dynamics. Incorporating both spatial heterogeneity and phenotypic adaptation may therefore be important for the consistent evaluation and design of fractionation strategies in heterogeneous tumors.

**Supplementary Information:**

The online version contains supplementary material available at https://doi.org/10.1007/s11538-026-01758-5.

## Introduction

Solid tumors are widely recognized as heterogeneous and evolving systems, in which malignant cells interact dynamically with their microenvironment (Hanahan and Weinberg 2000). Among microenvironmental factors, oxygen availability plays a central role in shaping both tumor progression and response to therapy.

Radiotherapy represents a standard treatment modality (Siegel et al. 2023), whose efficacy is strongly modulated by local oxygen levels. Oxygen enhances radiation-induced DNA damage, an effect quantified through the Oxygen Enhancement Ratio (OER), defined as the dose increase required under hypoxia to match the biological effect observed in normoxia.

However, oxygen influences treatment response in more than one way. Beyond its direct physico-chemical role in damage fixation, hypoxia induces transcriptional and metabolic reprogramming, promoting aggressive phenotypes that contribute to radioresistance (Beckers et al. 2024). Importantly, experimental studies indicate that OER is not uniform across intratumoral subpopulations. More aggressive cells are characterized by lower intrinsic radiosensitivity (Lagadec et al. 2012).

Moreover, oxygen distribution within the tumor microenvironment (TME) is typically spatially heterogeneous, owing to the irregular arrangement and perfusion of the tumor vasculature. As a consequence, not only the overall level of oxygenation, but also the geometry of oxygen sources and the resulting spatial gradients may contribute to shaping local radiotherapy response (Hormuth et al. 2021).

Mathematical models of tumor response to radiotherapy combine population growth dynamics with radiobiological descriptions of radiation-induced cell killing. Tumor kinetics are typically represented through proliferation laws incorporating carrying-capacity effects (Zheng et al. 2025), whereas radiation response is described by the linear–quadratic (LQ) formalism, in which the surviving fraction after a dose *d* is $S(d)=\exp (-\alpha d - \beta d^2)$, with α and β denoting radiosensitivity parameters inferred from experimental and clinical studies (van Leeuwen et al. 2018). The LQ formulation also underlies the definition of biologically effective dose (BED), enabling comparisons across fractionation schedules.

Experimental and clinical evidence on hypoxia led to extensions of the classical LQ model incorporating oxygen dependence. In particular, radiosensitivity parameters have been rescaled through an OER factor to account for variations in radiation response between normoxic and hypoxic conditions (Wenzl and Wilkens 2011). Alternative approaches have relaxed the assumption of homogeneous radiosensitivity by allowing the parameters α and β to vary across tumor cell populations or between patients, thereby representing intrinsic biological heterogeneity (Alfonso and Berk 2019).

Trait-structured models have recently been employed to describe tumor response to radiotherapy in the presence of both phenotypic heterogeneity and microenvironmental modulation (Celora et al. 2023; Chiari et al. 2023). In these formulations, spatial dynamics and continuous phenotypic structure are coupled within a unified PDE system, so that tumor response under irradiation can be interpreted as an eco-evolutionary process in which selection emerges from the redistribution of cells across phenotypic states.

Several mathematical studies have explored alternative radiotherapy fractionation strategies (Prokopiou et al. 2015; Henares-Molina et al. 2017; Brüningk et al. 2021). To our knowledge, however, the combined impact of spatially heterogeneous oxygenation and continuous phenotypic adaptation on the comparative performance of treatment schedules has not been systematically investigated. In particular, several protocols currently adopted in clinical practice are considered optimal for tumors exhibiting specific cellular and microenvironmental features; however, a mechanistic modeling framework capable of explaining these observed treatment preferences as a consequence of such features is still lacking.

In this context, this study is motivated by the need to understand how the interplay between oxygen spatial structure, evolutionary selection of resistant phenotypes, and normal-tissue toxicity constraints may shape the landscape of admissible fractionation protocols.

Are there dose–interval combinations, beyond those commonly used in clinical practice, that may lead to improved treatment outcomes? How does spatial heterogeneity in oxygen delivery influence the relative performance of radiotherapy schedules? Are protocols identified under spatially uniform conditions sufficiently robust, or is detailed characterization of spatial oxygen heterogeneity required for reliable treatment selection?

To address these questions, we develop a phenotypically structured partial differential equation (PS-PDE) model (Lorenzi et al. 2025). Within this framework, we systematically explore the dose–interval plane under biologically effective dose constraints, enabling comparison of fractionation protocols across different oxygenation regimes and tissue tolerance conditions. This modeling strategy aligns with current efforts toward biologically informed radiotherapy design, including multi-omics–guided treatment adaptation (Boldrini et al. 2024) and spatially targeted dose painting (Bentzen and Grégoire 2011).

The remainder of the paper is organized as follows. Section 2 introduces the mathematical model, including tumor spatio–phenotypic dynamics, oxygen dynamics, and the oxygen- and phenotype-dependent formulation of radiotherapy response. Section 3 investigates how oxygenation affects the performance of fractionation schedules under a common toxicity constraint, first under spatially uniform oxygen supply and then under heterogeneous oxygen delivery. Particular attention is given to the role of phenotypic adaptation and to how the spatial organization of oxygen supply can alter time-to-progression and protocol ranking. Conclusions and perspectives are discussed in Sect. 4.

## Methods

We introduce a spatio–phenotypic model coupling tumor-cell dynamics, oxygen diffusion–consumption, and oxygen- and phenotype-dependent radiotherapy response. Model parameters are listed in Table 1. Simulation details and indicator definitions are reported in the Supporting Information S1 and S2.

**Table 1:** Model parameters. Background colors distinguish parameter groups associated with cell dynamics (blue), radiotherapy response (red), and oxygen kinetics (green). The final column reports the origin of each parameter value, indicating whether it is taken from the literature, derived in a specific section of the paper, or estimated within the present framework as a model estimate (M.E.). Entries labeled with “S” refer to the corresponding section of the Supporting Information

### Cell Dynamics

We consider a portion of biological tissue where the early growth of a tumor mass occurs. The spatio–temporal evolution of the active cancer cell population is described by a density function *n*(*t*, *x*, *u*), where t denotes time, $x \in \Omega _s \subset \mathbb {R}$ represents the spatial position, and $u \in \Omega _p = [0,1]$ is a continuous phenotypic trait. Although spatial dynamics are modeled along a single coordinate, *n*(*t*, *x*, *u*) is interpreted as a volumetric cell density. Following the framework introduced in Chiari et al. (2023), we model resistance as a continuous variable ranging from fully hypoxia- and radiation-sensitive cells (u=0) to fully resistant ones (u=1), with higher values of u corresponding to increased resistance to both stresses. We emphasize that u represents an intrinsic phenotypic adaptation state, shaped by selection and non-genetic phenotypic changes, rather than a marker of a cell’s current, instantaneous oxygen exposure. A cell with high u therefore represents a phenotype adapted to hypoxic stress and intrinsically more resistant to radiation, consistently with the well-established association between hypoxia-induced cellular adaptation and radioresistance (Beckers et al. 2024).

The spatio–phenotypic avascular evolution of the tumor cell density is modeled through a Phenotypically Structured Partial Differential Equation (PS-PDE):

1 $$\begin{aligned} \partial _t n(t,x,u) = D_p\,\partial _u^2 n(t,x,u) + D_s\,\partial _x^2 n(t,x,u) + \bigl [R(t,x,u)-T(t,x,u)\bigr ]\,n(t,x,u) \end{aligned}$$

where $D_p$ represents the coefficient of phenotypic diffusion, accounting for stochastic epigenetic changes. The term $D_s$ corresponds to the linear spatial diffusion coefficient, which describes the random dispersal of cells within the tissue.

The function R(t,x,u) defines the net proliferation rate of the tumor cell population, whereas T(t,x,u) quantifies the effective rate of cell death induced by radiotherapy. Both terms depend on time, space, and phenotype, reflecting the heterogeneous nature of the tumor response to oxygen availability and radiation exposure.

#### Reaction Term

The proliferation rate R(t,x,u) models cell growth as influenced by oxygen availability, phenotypic adaptation, and crowding effects:

2 $$\begin{aligned} R\bigl (t,x,u\bigr ) = r_0 + \gamma _O\,M\bigl (O(t,x)\bigr )\,(1-u^2) - \gamma _H\bigl (1-M\bigl (O(t,x)\bigr )\bigr )\,(1-u)^2 - \kappa \,\rho (t,x) \end{aligned}$$

where $r_0$, $\gamma _O$, $\gamma _H$, and κ are model parameters, and $O(t,x):[0,t_{end}]\times \Omega _s\rightarrow \mathbb {R}_{+}$ denotes the local oxygen concentration.

The first term $r_0$ represents the baseline proliferation rate of fully resistant cells (u=1), which sustain a minimal growth rate even under adverse environmental conditions.

The second term provides an additional proliferative contribution, weighted by two effects: (i) a Hill-type function enhancing proliferation under normoxic conditions,

3 $$\begin{aligned} M\bigl (O(t,x)\bigr ) = \frac{O(t,x)^4}{O(t,x)^4+(\alpha _O)^4} \end{aligned}$$

and (ii) a quadratic dependence on u, expressing that phenotypes less resistant to radiotherapy and less adapted to hypoxia (u→0) exhibit higher proliferative potential.

This formulation reflects an energetic trade-off, whereby cells investing metabolic resources in resistance to hypoxia or radiation damage proliferate less efficiently.

The Hill exponent is set to $n_H=4$ as a modeling choice, providing a sharp but smooth transition and an effectively saturated oxygen-dependent proliferative contribution within the normoxic range. This value is not intended to represent a specific molecular cooperativity mechanism. The robustness of the results presented below with respect to this choice is assessed in Supporting Information S10 by repeating the analysis for different Hill coefficients.

The third term penalizes poorly adapted phenotypes under low-oxygen conditions, thereby acting as a selective pressure toward higher-resistance traits. The parameter $\gamma _H$ is set to $\gamma _H = r_0+\gamma _O$, so that under extreme hypoxia the penalty experienced by poorly adapted phenotypes offsets the largest intrinsic growth rate attained under favorable oxygenation.

The quadratic functional form in both the second and third terms is chosen so as to induce a concave fitness landscape: for fixed t and x, and depending on the oxygen concentration, the reaction term admits a unique global maximum with respect to the phenotypic variable u, as discussed in Supporting Information S3. This modeling assumption is consistent with analogous choices adopted in the literature on PS-PDEs (Lorenzi et al. 2025).

Finally, the crowding term enforces population saturation due to spatial limitations. Here, ρ(t,x) denotes the total local cell density,

4 $$\begin{aligned} \rho (t,x) = \int _{\Omega _p} n(t,x,u)\,du. \end{aligned}$$

For fixed (*t*, *x*, *u*), the net proliferation rate *R*(*t*, *x*, *u*) is decreasing with respect to the local density ρ, thereby limiting local tumor growth as cell density increases. The crowding coefficient κ is calibrated in Supporting Information S4 by prescribing K as an upper-bound carrying capacity under the most favorable proliferative conditions.

Overall, the reaction term defines a dynamic fitness landscape that evolves in time with both the oxygen distribution and the phenotypic composition of the tumor population.

### Radiotherapy Term

Radiation-induced cell death is modeled through a phenotype- and oxygen-dependent extension of the linear–quadratic (LQ) formulation. We write

5 $$\begin{aligned} T(t,x,u) = \bigl [\alpha (O(t,x),u)\, d + \beta (O(t,x),u)\, d^2 \bigr ]\, \delta _{\mathcal {T}}(t), \end{aligned}$$

where *d* denotes the dose delivered per fraction (Gy), and $\delta _{\mathcal {T}}(t)$ is a sum of Dirac delta functions that localize irradiation at prescribed treatment times, as detailed below.

The coefficients α(O,u) and β(O,u) are continuous counterparts of the classical constant LQ parameters and determine the survival fraction $S(d)=\exp \bigl (-T(t,x,u)\big )$ in the standard formulation (McMahon 2018). In our model they depend on both oxygen concentration and phenotype:

6 $$\begin{aligned} \begin{aligned} \alpha (O,u)&= \dfrac{\tilde{\alpha } + \Delta _\alpha (1-u)^2}{\textrm{OER}(O)} \\ \beta (O,u)&= \dfrac{\tilde{\beta } + \Delta _\beta (1-u)^2}{\textrm{OER}^2(O)} \end{aligned} \end{aligned}$$

The quadratic dependence on (1-u) introduces phenotype-specific modulation of radiosensitivity, consistently with patient-specific modeling studies showing that higher proliferation rates are associated with increased radiosensitivity (Rockne et al. 2010).

The OER formulation effectively rescales the delivered dose, so that $d/\textrm{OER}(O)$ represents an oxygen-dependent effective dose, with the quadratic term scaling accordingly as $d^2/\textrm{OER}^2(O)$.

The values of $\tilde{\alpha }$, $\tilde{\beta }$, $\Delta _{\alpha }$, and $\Delta _{\beta }$ are reported in Table 1. Further details on their derivation are provided in Supporting Information S5, where we also explore in depth the rationale underlying Eq. (6) and the theoretical implications of this formulation, which are used throughout the results presented in the following sections.

In addition to this intrinsic phenotypic contribution, radiosensitivity is further modulated by the local oxygen concentration: increasing oxygen availability enhances radiation-induced killing through an OER-based rescaling of the LQ parameters α(O,u) and β(O,u). We adopt the following functional form

7 $$\begin{aligned} \textrm{OER}(O) = 1 + \frac{(\textrm{OER}_{\max }-1)\, K_O}{\textrm{OER}_{\max }\,O + K_O} \end{aligned}$$

in line with the saturation law proposed by Howard-Flanders and Alper (1957). Since the OER rescales the effective delivered dose as $d/\textrm{OER}(O)$, the reciprocal factor $1/\textrm{OER}(O)$ is the quantity that increases with oxygenation in the radiation-response term. This factor ranges from $1/\textrm{OER}_{\max }$ under anoxic conditions (O→0) to 1 under well-oxygenated conditions $(O\rightarrow +\infty )$. The parameter $K_O$ corresponds to the oxygen level at which $1/\textrm{OER}(O)$ reaches the midpoint between its anoxic limit $1/\textrm{OER}_{\max }$ and its well-oxygenated limit 1.

Radiotherapy protocols are characterized by equally spaced isodoses and are denoted throughout by the triple $\mathcal {P}=(d,\Delta ,N_f)$, where *d* is the dose per fraction, Δ the inter-fraction interval, and $N_f$ the total number of fractions. If treatment starts at time $t^{RT}_0$, the set of irradiation times is $\mathcal {T}=\{t^{RT}_0+j\Delta \;|\; j=0,\dots ,N_f-1\}$.

The formulation in Eq. (5) is equivalent to imposing an instantaneous update of the cell density at each irradiation time $t^{RT}_i$:

8 $$\begin{aligned} n(t^{RT,+}_i,x,u) = S(d)\, n(t^{RT,-}_i,x,u), \end{aligned}$$

where $t^{RT,-}_i$, $t^{RT,+}_i$ denote instants immediately before and after dose delivery.

Cells lethally damaged by radiation are assumed to be removed instantaneously. Delayed effects (e.g., senescence, necrotic accumulation, and radiation-induced microenvironmental remodeling) as well as sublethal damage repair are not explicitly modeled; accordingly, corrections such as the Lea-Catcheside protraction factor (Brenner 2008) are not included.

### Oxygen Dynamics

The spatio–temporal evolution of the oxygen concentration *O*(*t*, *x*) is described by the following diffusion–consumption equation:

9 $$\begin{aligned} \partial _t O(t,x) = D_O\,\partial _{x}^2 O(t,x) - \lambda _O\,O(t,x) - \zeta _O\,\rho (t,x)\,O(t,x) + V_O(x) \end{aligned}$$

Here, $D_O$ denotes the oxygen diffusion coefficient in the tissue, and a Fickian diffusion process is assumed for simplicity.

The term $-\lambda _O O$ accounts for physiological oxygen consumption by healthy tissue at reference density.

Oxygen consumption by tumor cells is modeled through the nonlinear term $-\zeta _O \rho O$.

The consumption rate is assumed phenotype-independent for simplicity, as the present framework does not explicitly resolve alternative metabolic pathways or phenotype-dependent oxygen-consumption mechanisms.

Since the previous term already accounts for oxygen consumption by healthy tissue at physiological density, the tumor contribution is interpreted as an effective excess uptake associated with the replacement of healthy cells by tumor cells.

The oxygen variable O(t,x) is expressed throughout the paper in oxygen partial-pressure units (mmHg). Parameter values for $\lambda _O$ and $\zeta _O$ are reported in Table 1, and further details on their calibration are provided in Supporting Information S6. These parameters are chosen to ensure that oxygen dynamics rapidly reach quasi-steady conditions relative to the slower timescale of cell population dynamics.

Finally, the source term $V_O(x)$ represents oxygen supply from the vasculature. Allowing $V_O$ to depend on space enables the modeling of heterogeneous vascular structures, ranging from simplified and controlled configurations (e.g., *in vitro* conditions) to more complex and spatially irregular oxygen inputs characteristic of *in vivo* tumor environments. In this work, $V_O$ is assumed to be time-independent for the sake of model simplicity, since vascular dynamics are not explicitly modeled and lie outside the scope of the present study.

## Results

We use the model to compare radiotherapy schedules across oxygenation regimes, using time-to-progression as the primary outcome. We first analyze spatially uniform oxygen supply to characterize the dose–interval response landscape and its mechanistic drivers, and then examine how toxicity constraints, intermediate oxygenation levels, and spatial oxygen-source heterogeneity reshape protocol ranking.

### Schedule Ranking under Uniform Oxygenation

A natural starting point for applying the model is to consider tumor evolution under a spatially uniform oxygen supply.

In the absence of tumor cells (ρ=0) and under spatially uniform conditions, the steady state for the oxygen equation (9) is given by $O^* = V_O/\lambda _O$. In the following, we fix the tumor-free steady state to a reference oxygenation level $I_0$, with $I_0 \in \{O_M, O_m, O_h\}$ (see Table 1), which determines the oxygen supply as $V_O(x) = I_0 \lambda _O$ for all $x \in \Omega _s$, yielding a homogeneous and constant oxygen input.

From a biological perspective, this setting can be interpreted as tumor evolution occurring near a vascular structure, such as a single vessel or a cluster of vessels, resulting in an approximately uniform oxygen supply along the spatial direction under consideration and constant over time.

Under this configuration, oxygen rapidly becomes nearly uniform within the tumor core, with only small boundary-layer deviations, owing to its fast diffusion relative to the timescale of cell dynamics.

For this reason, this scenario serves as a reference configuration before considering more complex spatial oxygen distributions.

Normal tissues differ in their sensitivity to fraction size, which in the LQ framework is characterized by the $(\alpha /\beta )_H$ ratio. Protocol comparisons are therefore performed under a fixed constraint on the biologically effective dose (BED), defined in Supporting Information S2, by requiring that the BED of each admissible protocol does not exceed that of a conventional standard-of-care (SoC) schedule $\mathcal {P}_{SoC}=(d_{SoC}=1.8~\textrm{Gy}, \Delta _{SoC}=1~\textrm{day}, N_{f,SoC}=30)$, namely

10 $$\begin{aligned} \textrm{BED}(\mathcal {P}) \le \textrm{BED}(\mathcal {P}_{SoC}) . \end{aligned}$$

For each prescribed dose per fraction *d*, the number of fractions is chosen as the largest integer compatible with this constraint,

11 $$\begin{aligned} N_f= \left\lfloor \frac{\textrm{BED}(\mathcal {P}_{SoC})}{d\left( 1+d/(\alpha /\beta )_H\right) } \right\rfloor . \end{aligned}$$

Equivalently, among all integer numbers of fractions satisfying the BED constraint, we select the one that yields the largest BED not exceeding $\textrm{BED}(\mathcal {P}_{SoC})$. Thus, for each dose–interval pair (d,Δ), the protocol $\mathcal {P}=(d,\Delta ,N_f)$ is uniquely specified. This construction enforces comparable levels of healthy-tissue toxicity across treatment schedules. By preventing an artificial increase in the effective dose, it allows the model to test the effects of fractionation and temporal scheduling separately from trivial dose escalation.

Following the setup proposed by Henares-Molina et al. (2017), we explore alternative radiotherapy schedules by varying the dose per fraction *d* and the inter-fraction interval Δ. Calendar effects associated with clinical delivery schedules, such as weekend breaks, are not explicitly considered in the present model.

The explored dose range is $d \in (0,5]\,\textrm{Gy}$, consistent with the validity limits of the LQ model, which is known to lose accuracy beyond this interval (Kirkpatrick et al. 2008; Cui et al. 2022). Time intervals are taken as $\Delta \in (0,100]$ days, again following Henares-Molina et al. (2017).

This extended interval range is introduced to reveal the structural dependence of treatment outcome on the dose–interval trade-off. Although many of the explored intervals exceed typical clinical practice, they are included to characterize the underlying response behavior. As shown below, slices of the treatment landscape along either the dose or interval axis retain a similar qualitative structure, with systematic shifts in response, indicating a robust correlation between fraction spacing and dose rather than isolated optima.

**Fig. 1:**
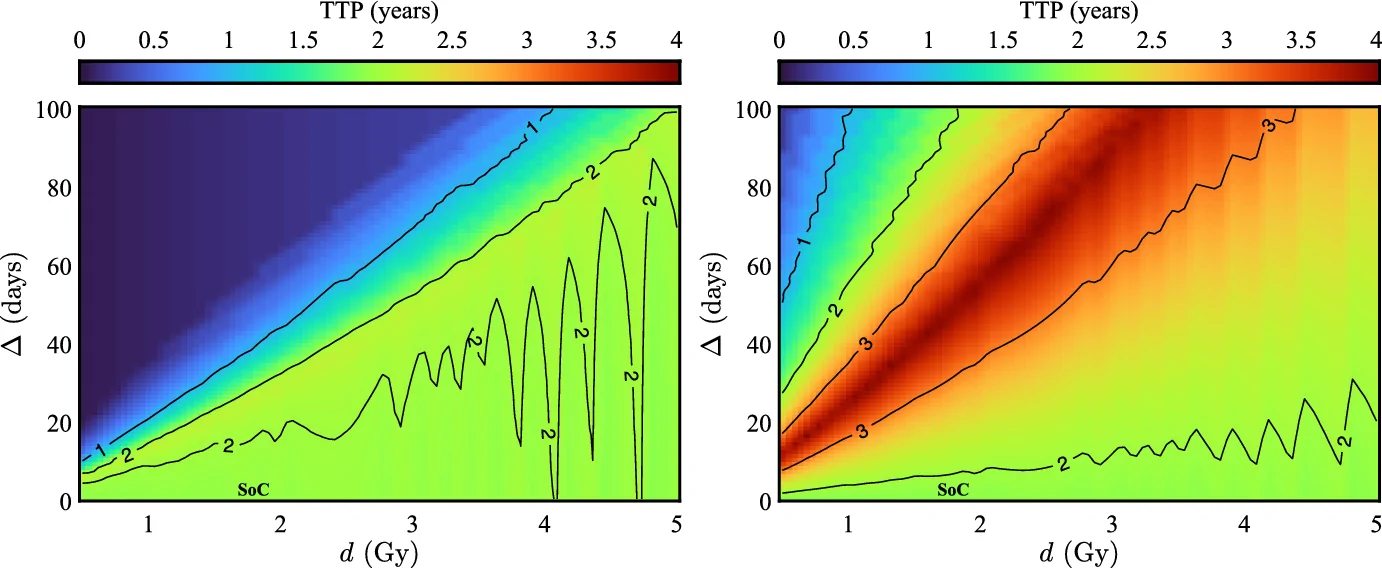
Time-to-progression (TTP), measured in years, as a function of single-fraction dose and inter-fraction interval under a spatially uniform oxygen supply. **Left:** normoxic conditions ($I_0=O_M$). **Right:** hypoxic conditions ($I_0=O_m$). Protocol comparisons are performed under the constraint $\textrm{BED}(\mathcal {P}) \le \textrm{BED}(\mathcal {P}_{\textrm{SoC}}) \approx 64\,\textrm{Gy}$, where the standard-of-care protocol $\mathcal {P}_{\textrm{SoC}}$ consists of 30 fractions of $1.8\,\textrm{Gy}$ delivered at 1-day intervals. Healthy-tissue fractionation sensitivity is characterized by $(\alpha /\beta )_H = 10\,\textrm{Gy}$, consistent with values reported in Henares-Molina et al. (2017). Oxygenation qualitatively reshapes the dose–interval response landscape: under normoxia, protocol performance is relatively insensitive to fractionation choice, whereas under hypoxia, protracted schedules with longer inter-fraction intervals achieve substantially greater TTP than the standard-of-care schedule

Figure 1 displays the predicted time-to-progression (TTP) across the (d,Δ) parameter space under spatially uniform oxygen supply, in normoxic (left panel) and hypoxic (right panel) conditions. Here, TTP denotes the time elapsed from the initiation of radiotherapy to tumor relapse, defined as the time at which the total tumor burden returns to the prescribed detection threshold; its formal definition is reported in Supporting Information S2. Each dose–interval map was obtained through a systematic scan of the protocol space based on 2500 independent model runs. Each run corresponds to a single dose–interval pair (d,Δ), for which the number of fractions is determined by the BED constraint and the resulting TTP is recorded.

Under high oxygenation, the TTP landscape is relatively flat, with a broad region of dose–interval combinations yielding comparable outcomes. In this regime, the predicted time-to-progression remains close to the value obtained under the SoC schedule, typically around two years, and deviations from this reference produce only marginal variations in relapse timing.

In contrast, under hypoxic conditions the structure of the map changes qualitatively. A distinct region of the parameter space emerges in which TTP values increase substantially, reaching almost four years along a well-defined ridge in the (d,Δ) plane. This structure delineates a *therapeutic efficiency frontier*, separating protocols with limited benefit from schedules capable of nearly doubling the time-to-progression relative to standard fractionation. Notably, the frontier is located in the region of the map characterized by longer inter-fraction intervals, indicating that more protracted or metronomic-like fractionation schedules can achieve the largest TTP gains under hypoxic conditions.

The linear-like geometry of this high-TTP region further indicates the presence of a persistent trade-off between dose per fraction and inter-fraction spacing: rather than a single isolated optimum, comparable outcomes can be achieved along a continuous band of dose–interval combinations in the (d,Δ) plane.

### Interplay between Reoxygenation and Phenotypic Selection

To qualitatively elucidate the mechanisms underlying the observed structure of the dose–interval map, we compare tumor dynamics generated by two representative protocols located in distinct regions of Fig. 1: the SoC schedule and a protocol attaining maximal TTP within the same map. These cases serve as prototypical examples of low- and high-performance regimes and illustrate the dynamical mechanisms shaping treatment response. The resulting insights are consistent with the broader analysis presented in Sect. 3.4, where we systematically explore a range of oxygenation levels and evaluate the distribution of gains across near-optimal protocols. This analysis reveals a non-monotone dependence of treatment benefit on oxygen availability, with intermediate hypoxia emerging as the regime in which fractionation and timing have the largest impact.

**Fig. 2:**
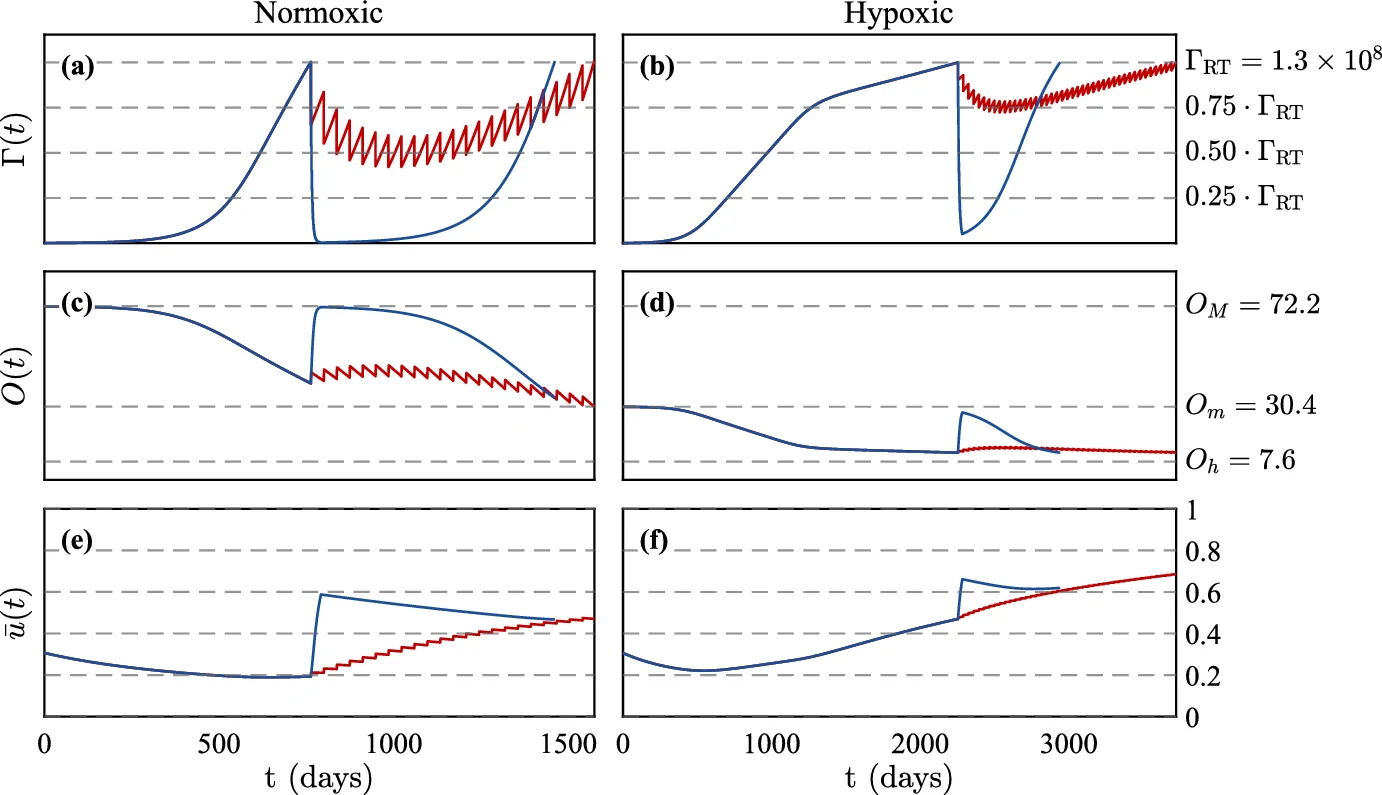
Time evolution of tumor burden (top), oxygen level (middle), and mean phenotypic trait (bottom) under the standard-of-care protocol (blue solid line) and the maximal-TTP protocol (red solid line). Panels (a,b) show tumor burden, panels (c,d) the oxygen level, and panels (e,f) the mean phenotypic trait. Tumor burden Γ(t) is expressed as the total number of cells, while oxygen levels and thresholds are expressed in $\textrm{mmHg}$. The standard-of-care protocol consists of 30 fractions of $1.8\,\textrm{Gy}$ delivered at 1-day intervals. The detection threshold $\Gamma _{\textrm{RT}}$ and the oxygenation thresholds $O_M$, $O_m$, and $O_h$ are reported in Table 1. Panels (a,c,e) correspond to the normoxic case $(I_0 = O_M)$: the maximal-TTP protocol delivers approximately $2.3\,\textrm{Gy}$ every 15 days for 22 fractions (total dose $\sim 51\,\textrm{Gy}$), yielding a TTP gain of approximately 113 days. Panels (b,d,f) correspond to the hypoxic case $(I_0 = O_m)$: the maximal-TTP protocol delivers approximately $1.3\,\textrm{Gy}$ every 35 days for 42 fractions (total dose $\sim 55\,\textrm{Gy}$), yielding a TTP gain of approximately 2 years. Treatment-induced reoxygenation interacts differently with phenotypic selection across oxygenation regimes: under normoxia, SoC-induced reoxygenation is partially offset by redistribution toward resistant phenotypes, whereas under hypoxia resistance is already established, leading to earlier relapse under SoC than under the protracted maximal-TTP schedule (Color figure online)

Figure 2 shows the time evolution of tumor burden, oxygen concentration, and mean phenotype (see Supporting Information S2 for its definition) for both protocols under normoxic and physiological hypoxic conditions.

During the initial stage, before treatment delivery, the time evolution of the three reported quantities coincides for the two protocols, since tumor growth and the associated microenvironmental pressure are identical. Differences emerge only after radiation delivery, when the trajectories associated with the two protocols start to diverge. In the hypoxic case (panels (b,d,f) of Fig. 2), the visible change in slope of the pre-treatment tumor-burden curve around t≈1000 days reflects a progressive slowing of tumor growth as the population expands. Increasing local cell density strengthens the crowding contribution in Eq. (2), while the accompanying decrease in oxygen availability further limits proliferation and promotes selection toward more hypoxia-adapted, less proliferative phenotypes. These combined effects reduce the net growth rate and explain the longer time required to reach the detection threshold $\Gamma _{\textrm{RT}}$ compared with the normoxic case.

As shown in panels (a,c,e) of Fig. 2, under normoxic conditions, the maximal-TTP protocol operates within a mildly deoxygenated TME and induces only a limited phenotypic shift. In contrast, the SoC protocol promotes tumor reoxygenation while delivering more densely spaced radiation fractions, thereby redistributing the population toward higher-resistance trait values. As a result, microenvironmental effects and therapy-induced selection partially offset one another, leading to comparable TTP values despite markedly different underlying dynamics.

Under hypoxic conditions (panels (b,d,f) of Fig. 2), the maximal-TTP protocol again maintains low oxygenation levels with only minor changes in the phenotypic distribution. The SoC protocol still increases oxygen availability; however, unlike the normoxic case, this does not induce a substantial redistribution of phenotypes. In this regime, the TME has already selected for highly resistant phenotypes, leaving little room for further therapy-driven phenotypic switching. Consequently, the balance observed under normoxia disappears: resistance is already established, resulting in earlier relapse of the SoC protocol after reoxygenation.

### Impact of Toxicity Constraints

So far, protocol comparisons have been performed under the constraint that the equivalent toxicity does not exceed that of a SoC schedule. This assumption, however, is not universally appropriate. In some clinical scenarios, dose escalation may be considered to improve tumor control (Brower et al. 2016), whereas in others stricter toxicity limits may require a reduction of the admissible dose (Timmerman et al. 2006).

From a clinical standpoint, treatment planning is inherently multi-objective, involving a trade-off between tumor control probability (TCP) (Gong et al. 2013) and normal-tissue toxicity, commonly quantified through the Normal Tissue Complication Probability (NTCP) (Gaito et al. 2022). Additional variability arises from patient- and site-specific factors, including intrinsic radiosensitivity (Nuijens et al. 2025) and the geometric configuration of organs at risk (OARs) (Eber et al. 2025). These considerations suggest that differences in normal-tissue responsiveness and tolerance to fraction size modulate the feasible treatment space and should therefore be incorporated into protocol comparison.

The aim of the following analysis is to characterize how protocol efficiency depends on oxygen availability, normal-tissue radiosensitivity, and admissible toxicity levels.

To this end, we extend the exploration of simulated radiotherapy schedules by considering multiple combinations of BED, normal-tissue radiosensitivity $(\alpha /\beta )_H$, and oxygen availability.

TTP is again adopted as the efficacy metric. The therapeutic efficiency frontier – formally defined in Supporting Information S7 – is identified as the subset of dose–interval combinations attaining at least 80% of the maximum predicted TTP.

For each parameter configuration, we compute a TTP-weighted barycenter of the frontier (see Supporting Information S7 for details). This barycenter is not intended to identify an optimal schedule; rather, it provides a compact descriptor of the region in the dose–interval plane associated with highly effective regimens, thereby enabling systematic comparison across oxygenation and toxicity scenarios.

Nine parameter configurations are analyzed by combining three oxygenation states—normoxic $(I_0 = O_M)$, hypoxic $(I_0 = O_m)$, and severely hypoxic $(I_0 = O_h)$—with three paired toxicity settings: $(\textrm{BED},(\alpha /\beta )_H)=(90\,\textrm{Gy},10\,\textrm{Gy})$, $(60\,\textrm{Gy},6\,\textrm{Gy})$, and $(30\,\textrm{Gy},2\,\textrm{Gy})$.

**Fig. 3:**
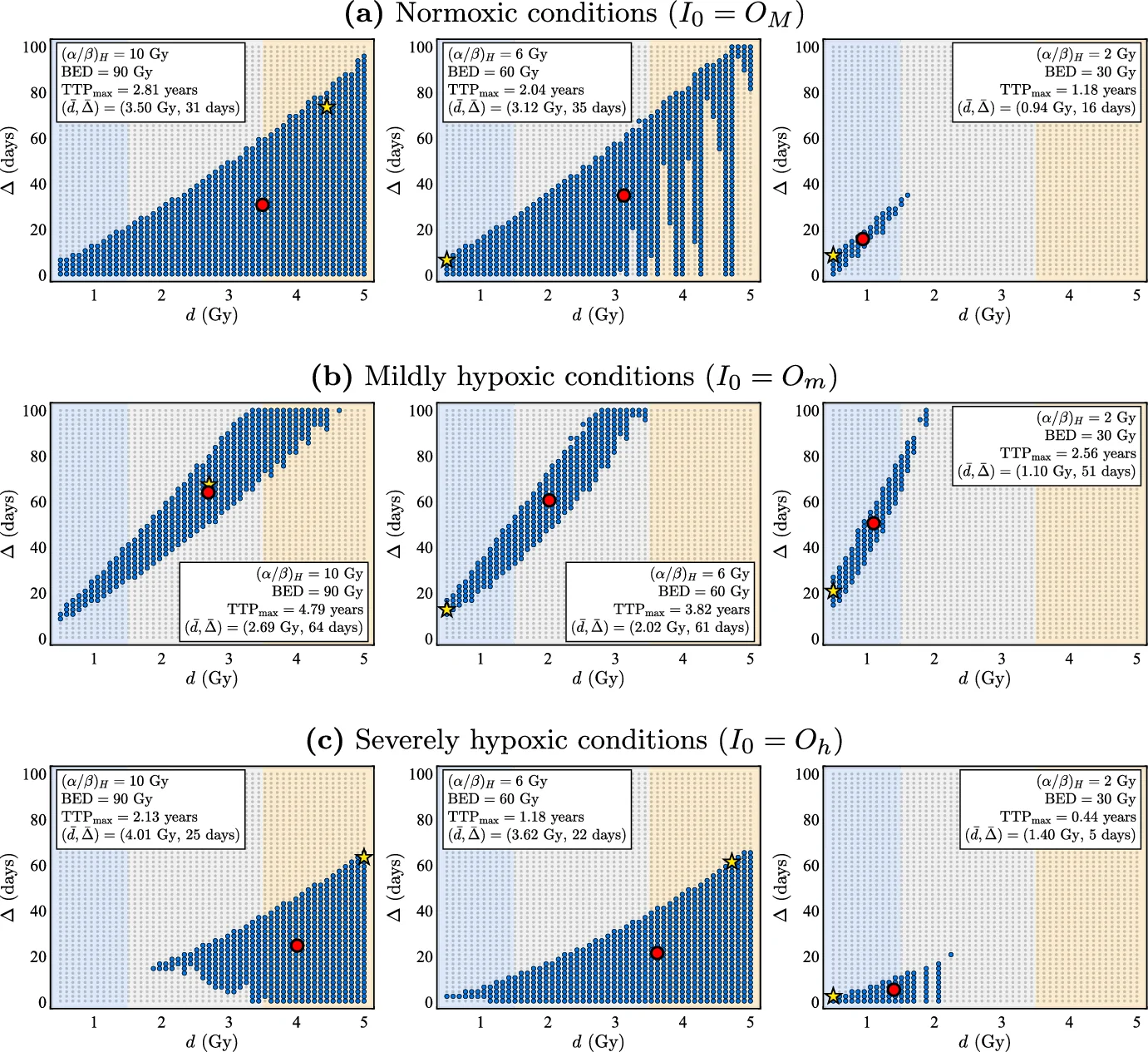
Therapeutic efficiency frontiers in the dose–interval plane under spatially uniform oxygen supply. Each column corresponds to a fixed normal-tissue sensitivity $(\alpha /\beta )_H$ and BED constraint, common to all oxygenation regimes shown in that column. Rows correspond to increasing hypoxia: **(a)** normoxic conditions $(I_0=O_M)$, **(b)** moderately hypoxic conditions $(I_0=O_m)$, and **(c)** severely hypoxic conditions $(I_0=O_h)$. For each parameter configuration, the therapeutic efficiency frontier contains the protocol combinations achieving at least 80% of the maximum TTP. Star markers indicate the dose–interval pair achieving maximal TTP, while circular markers indicate the corresponding TTP-weighted barycenters. The dose–interval plane is partitioned into three regions to distinguish schedules typically classified as hyperfractionated ($d \le 1.5\,\textrm{Gy}$), standard-to-moderately hypofractionated ($1.5\,\textrm{Gy}< d < 3.5\,\textrm{Gy}$), and strongly hypofractionated ($3.5\,\textrm{Gy} \le d \le 5\,\textrm{Gy}$). Oxygenation and normal-tissue tolerance jointly reshape the therapeutic efficiency frontier. Protracted schedules are favored under moderate hypoxia, whereas under severe hypoxia hypofractionated schedules can be advantageous at higher tolerance levels

Under normoxic conditions (Fig. 3a), the therapeutic efficiency frontier spans a broad region of the dose–interval plane, reflecting the limited sensitivity of TTP to protocol variations in this regime. A wide range of schedules yields comparable outcomes, consistent with the relatively flat TTP landscape observed under uniform oxygenation. As toxicity constraints become more restrictive, the admissible region narrows and the frontier barycenter shifts toward more hyperfractionated schedules.

As oxygen availability decreases, the therapeutic efficiency frontier exhibits a clear diagonal structure (Fig. 3b). The maximal predicted TTP also increases substantially relative to normoxic conditions, in some cases approximately doubling across the considered toxicity constraints. In this regime, longer inter-fraction intervals are favored, consistently with the mechanism discussed in Sect. 3.2: hypoxia-driven selection stabilizes slowly proliferating, hypoxia-adapted resistant phenotypes, weakens the compensatory effect of reoxygenation, and thereby reduces the benefit of closely spaced irradiation. Reduced normal-tissue tolerance narrows the diagonal and increases its slope. Since higher doses are no longer admissible, compensation occurs through longer inter-fraction intervals. In this configuration, the frontier is largely confined to protracted schedules over a broad range of admissible toxicity levels. The barycenter therefore lies in the metronomic region, indicating that such schedules remain robust across varying tissue tolerance conditions.

In severely hypoxic conditions (Fig. 3c), the therapeutic efficiency frontier no longer exhibits the regular diagonal structure observed at higher oxygen levels. In addition, the maximal achievable TTP decreases relative to the mildly hypoxic regime, reflecting the reduced effectiveness of radiation under extreme oxygen deprivation. In this setting protocol selection is primarily governed by normal-tissue tolerance. When tolerance is relatively high, hypofractionated schedules dominate the admissible region, whereas decreasing tolerance induces a transition toward hyperfractionated schedules, which become progressively accessible. Consistently, the barycenter shifts from hypofractionated to hyperfractionated regions of the dose–interval plane as toxicity constraints become more restrictive. This observation aligns with the dynamics of slowly proliferating, late-responding tumors and is consistent with clinical evidence showing that tumors such as prostate cancer may benefit from hypofractionated treatment schedules (Fowler et al. 2001).

This result provides a complementary perspective to standard radiobiological modeling by incorporating phenotypic structure. Classical LQ–OER formulations, which are largely empirical and do not account for phenotypic heterogeneity, typically associate hypoxia with an increased $\alpha /\beta$ ratio, thereby reducing the predicted benefit of hypofractionation. In contrast, our framework couples oxygenation with phenotypic selection, so that the $\alpha /\beta$ ratio emerges as a dynamic quantity. As detailed in Supporting Information S5, this mechanism can lead to a decrease of the $\alpha /\beta$ ratio under hypoxia, consistent with clinical observations and enabling the emergence of hypofractionation-favorable regimes.

### Extension to Intermediate Oxygenation Levels

We next refine the uniform-oxygenation analysis by considering a denser set of oxygen supply levels between pathological hypoxia and normoxia, $I_0\in [O_h,O_M]$. For each value of $I_0$, we reconstructed the dose–interval TTP map and selected the corresponding therapeutic efficiency frontier, using the same definition as in Supporting Information S7. In particular, the frontier identifies the subset of dose–interval combinations that achieve near-maximal TTP values under the imposed constraints, thereby capturing the region of high-performing treatment protocols. In this analysis, all protocols were generated under the BED constraint associated with the standard-of-care schedule $\mathcal {P}_{\textrm{SoC}}$, with healthy-tissue fractionation sensitivity $(\alpha /\beta )_H=10$ Gy, as introduced in Sect. 3.1.

For each oxygenation level, let $\mathcal {F}(I_0)$ denote the set of protocols belonging to the therapeutic efficiency frontier. We then evaluate how these near-optimal protocols perform relative to the standard-of-care schedule under the same oxygenation condition. To this end, for every protocol $\mathcal {P}\in \mathcal {F}(I_0)$, we define the TTP gain as

12 $$\begin{aligned} g_{\mathcal {P}}(I_0) = \textrm{TTP}_{\mathcal {P}}(I_0) - \textrm{TTP}_{\textrm{SoC}}(I_0). \end{aligned}$$

**Fig. 4:**
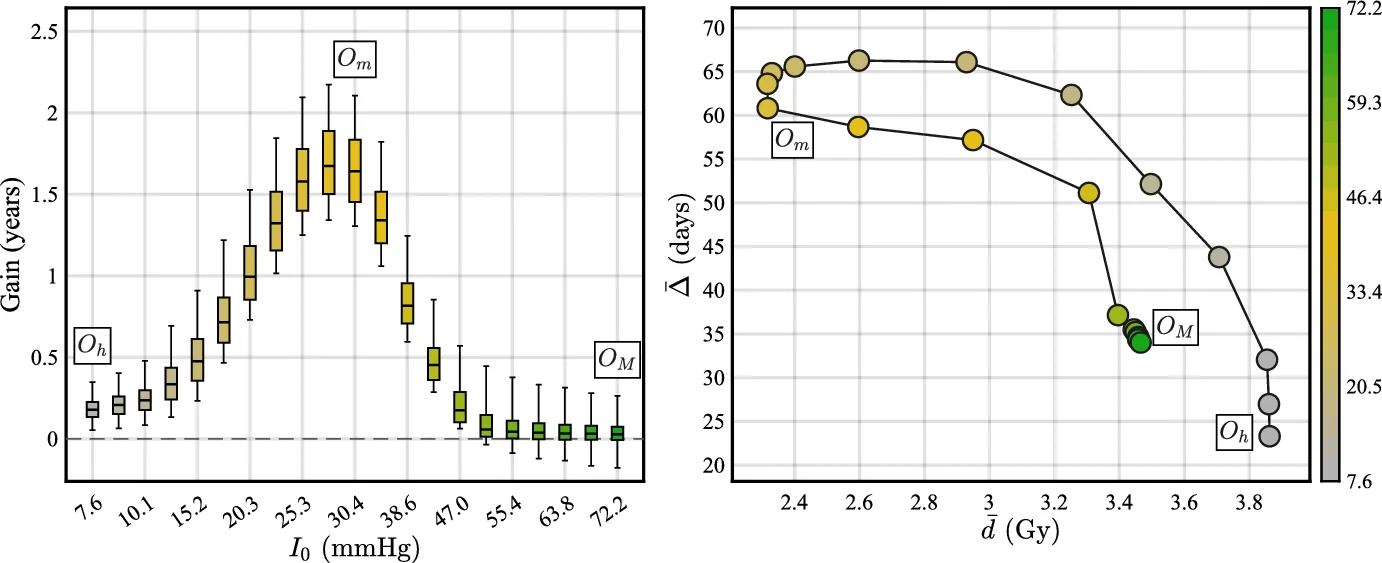
Extension of the uniform oxygenation analysis to intermediate oxygen supply levels. For each value of $I_0$, the dose–interval TTP map is reconstructed from 2500 independent model runs, and the therapeutic efficiency frontier is then extracted and analyzed. **Left:** distribution of TTP gains, measured relative to the standard-of-care protocol, over the corresponding efficiency frontier. Boxes indicate the interquartile range, central lines denote medians, and whiskers indicate minimum and maximum gains. Colors encode oxygenation level. The dashed line marks zero gain. **Right:** TTP-weighted barycenter of the same efficiency frontier in the dose–interval plane as a function of $I_0$. The benefit of departing from standard-of-care fractionation is non-monotone in oxygen availability, peaking at intermediate hypoxia and becoming limited under both normoxia and severe hypoxia

The distribution of $g_{\mathcal {P}}(I_0)$ over $\mathcal {F}(I_0)$ was summarized by its median, minimum and maximum values, together with lower and upper quartile indicators. These quantities were used to construct the boxplot-like representation shown in Fig. 4 (left panel).

This analysis shows that the benefit of alternative fractionation schedules is not a monotone function of oxygen availability. In well-oxygenated conditions, the gain is negligible or only marginal, consistently with the relatively flat TTP landscape observed under normoxia. As oxygenation decreases, the gain increases and reaches its largest values in the intermediate hypoxic range. This identifies a window in which the standard-of-care schedule is no longer close to the most effective region of the dose–interval landscape. Under severe hypoxia, however, the gain decreases again. This non-monotone behavior suggests that moderate hypoxia is the regime in which fractionation and timing have the largest impact: oxygen is sufficiently low to reshape phenotypic selection and protocol ranking, but not so low that radiation efficacy is globally suppressed. This observation is consistent with the mechanistic picture outlined in Sect. 3.2, where the interplay between reoxygenation and phenotypic selection governs treatment response across oxygenation regimes.

Using the TTP-weighted barycenter introduced above as a compact descriptor of each frontier, Fig. 4 (right panel) provides a roadmap of how the high-performance region of the dose–interval plane shifts with $I_0$. In normoxic conditions, the frontier is centered on moderately hypofractionated schedules. As oxygenation decreases toward moderate hypoxia, the barycenter moves toward lower doses per fraction and longer inter-fraction intervals, corresponding to more protracted, metronomic-like schedules. In severe hypoxia, the trajectory bends back toward larger doses per fraction and shorter intervals, consistently with the behavior observed under the previous toxicity-constraint analysis.

Taken together, the two panels of Fig. 4 provide a consistent picture. The non-monotone shift of the frontier across oxygenation levels (right panel) is accompanied by a corresponding non-monotone trend in the gain distribution (left panel), with maximal improvements observed in the intermediate hypoxic range. In normoxia, gains remain limited, whereas in severe hypoxia the reduction in oxygen-mediated radiosensitization constrains the achievable benefit despite the shift toward larger fraction sizes. Overall, these results identify intermediate hypoxia as the regime in which deviations from standard fractionation are most consequential, with a systematic shift toward more protracted schedules.

### Role of Oxygen Spatial Heterogeneity

The assumption of spatially uniform supply neglects the intrinsic geometric heterogeneity of tumor vasculature. Spatial variations in oxygen concentration are expected to modify local radiosensitivity, reshape eco-evolutionary tumor dynamics, and ultimately alter protocol ranking.

To isolate this geometric effect, we shift our focus from the uniform configuration to an ensemble of spatially heterogeneous oxygen sources associated with the same reference tumor-free oxygenation level $I_0$. In the following, we consider two representative oxygenation regimes, corresponding to normoxic ($I_0 = O_M$) and moderately hypoxic ($I_0 = O_m$) conditions.

For each of these regimes, we generate multiple realizations of the oxygen source, each corresponding to a different spatial redistribution of the same total amount. In practice, each realization is obtained by combining a finite number of localized contributions with randomly sampled positions, amplitudes, and spatial extents, and then rescaled so as to preserve the same global oxygen input. This construction produces a broad range of spatial configurations, from nearly uniform profiles to strongly localized and polarized distributions. As a result, differences in treatment outcome can be attributed to the geometry of oxygen delivery rather than to changes in the overall oxygen supply. Details on the construction of these heterogeneous oxygen sources are provided in Supporting Information S8.

Under these conditions, identical radiotherapy schedules may produce substantially different times-to-progression, highlighting the role of microenvironmental geometry in determining relapse dynamics.

We then assess the robustness of protocol selection with respect to this spatial perturbation. The analysis is designed to separate two distinct steps: protocol selection under a simplified homogeneous assumption, and protocol evaluation under heterogeneous oxygen delivery. The procedure is applied independently to the two reference oxygenation regimes considered in this section, $I_0\in \{O_M,O_m\}$.

For each value of $I_0$, we first identify the therapeutic efficiency frontier $\mathcal {F}_u(I_0)$ under spatially uniform oxygen supply, constructed exactly as in Sect. 3.4. Thus, $\mathcal {F}_u(I_0)$ is the set of protocols that would be selected as near-optimal if only the corresponding uniform oxygen input were available.

We next test these same protocols on heterogeneous oxygen-source configurations, without redefining the frontier and without re-optimizing the schedules for each geometry. Specifically, for each value of $I_0$, we generate M=100 heterogeneous oxygen-source Monte Carlo realizations

13 $$\begin{aligned} V_{O,I_0}^{(m)}(x), \qquad m=1,\ldots ,M, \end{aligned}$$

all normalized to have the same total oxygen input as the corresponding uniform-source case. For each realization, we evaluate the same dose–interval grid used in the uniform analysis, consisting of 2500 protocols satisfying the BED constraint. Therefore, the heterogeneous-source analysis consists of 2500×100 simulations per oxygenation regime, and $2500\times 100\times 2$ simulations in total across the two regimes. These heterogeneous simulations are used to evaluate the performance of protocols selected from $\mathcal {F}_u(I_0)$, rather than to construct a new geometry-specific efficiency frontier.

For each value of $I_0$, each heterogeneous realization m, and each protocol $\mathcal {P}\in \mathcal {F}_u(I_0)$, we compute the gain

14 $$\begin{aligned} g_{\mathcal {P}}^{(m)}(I_0) = \textrm{TTP}_{\mathcal {P}}^{(m)}(I_0) - \textrm{TTP}_{\textrm{SoC}}^{(m)}(I_0), \end{aligned}$$

where both terms are evaluated under the same oxygen-source realization $V_{O,I_0}^{(m)}(x)$. This approach compares each protocol with the standard-of-care schedule within the same oxygenation regime and spatial configuration, so that the resulting gain measures the benefit of protocol choice rather than the absolute effect of a more or less favorable geometry.

To quantify the degree of spatial heterogeneity, each realization $V_{O,I_0}^{(m)}(x)$ is assigned a *Gini coefficient* $\mathcal {G}_{I_0}^{(m)}$, computed from the corresponding discretized oxygen-source profile. This index primarily captures the spatial polarization of oxygen supply, with zero Gini coefficient corresponding to the perfectly uniform source and larger values indicating increasingly localized oxygen delivery. We use the Gini coefficient as a compact descriptor of heterogeneity, although other spatial indicators could in principle capture complementary geometric features.

**Fig. 5:**
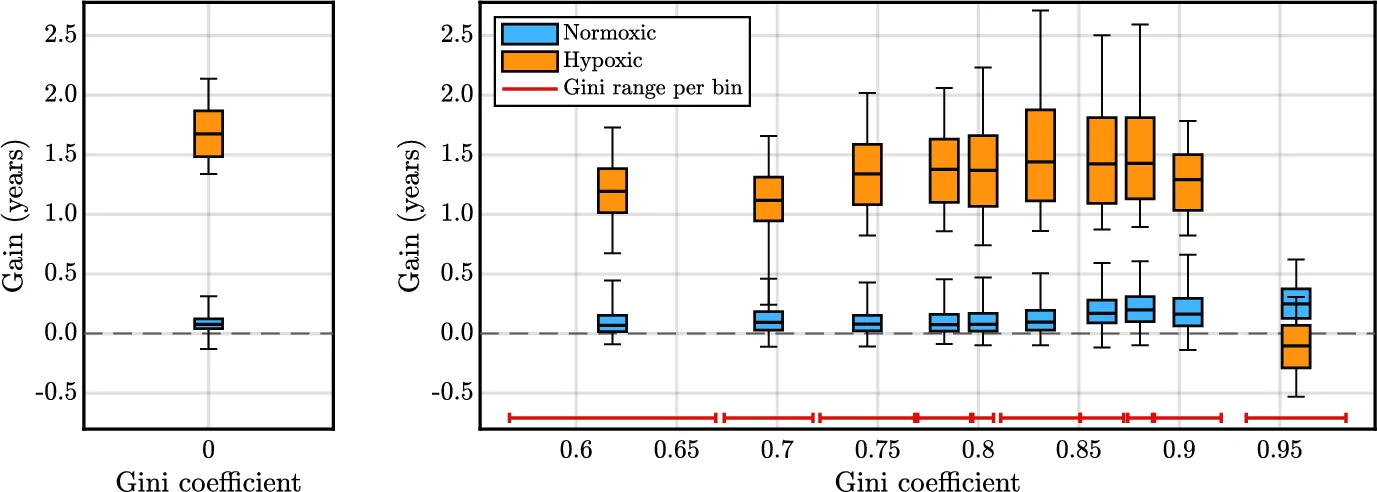
Effect of spatial oxygen-source heterogeneity on protocols selected under uniform-supply assumptions. For each $I_0\in \{O_M,O_m\}$, protocols belonging to the uniform-source frontier $\mathcal {F}_u(I_0)$ are evaluated under either the corresponding uniform source or heterogeneous oxygen-source configurations, without re-optimization. Gains are measured relative to the standard-of-care protocol under the same oxygenation regime and spatial configuration. **Left:** uniform-source reference cases, for which the Gini coefficient is zero. **Right:** heterogeneous configurations ordered by increasing Gini coefficient and grouped into bins of 10 realizations, separately for each $I_0$. Blue boxes indicate normoxic conditions ($I_0=O_M$); orange boxes indicate moderately hypoxic conditions ($I_0=O_m$). Boxes represent the interquartile range, central lines denote medians, and whiskers indicate minimum and maximum gains. Red segments indicate the Gini range of each bin, and the dashed line marks zero gain. Protocols selected as near-optimal under spatially uniform oxygenation remain qualitatively robust under heterogeneous oxygen delivery, but their individual benefit becomes increasingly variable and can turn negative as spatial heterogeneity increases, especially under hypoxia (Color figure online)

Figure 5 is constructed as follows. The left panel reports the uniform-source reference cases. For each oxygenation regime, the corresponding boxplot summarizes the distribution of gains obtained by protocols in $\mathcal {F}_u(I_0)$ under spatially uniform oxygen supply. The right panel reports the heterogeneous cases. For each value of $I_0$, the geometries are ordered by increasing $\mathcal {G}_{I_0}^{(m)}$ and grouped into consecutive bins of 10 geometries each. For each bin, the boxplot summarizes the pooled set of gains

15 $$\begin{aligned} \left\{ g_{\mathcal {P}}^{(m)}(I_0) : \mathcal {P}\in \mathcal {F}_u(I_0),\ m\in B_k(I_0) \right\} , \end{aligned}$$

where $B_k(I_0)$ denotes the k-th Gini bin for the oxygenation regime $I_0$. Boxes indicate the interquartile range, central lines denote medians, and whiskers indicate minimum and maximum gains. The horizontal red segment associated with each bin reports the corresponding Gini interval, from the minimum to the maximum $\mathcal {G}_{I_0}^{(m)}$ among the geometries in that bin. Thus, each box describes how the gain of protocols selected under uniform oxygenation varies across heterogeneous geometries with comparable Gini coefficient. The two series of boxplots correspond to the two total oxygen supplies $I_0=O_M$ and $I_0=O_m$. Several features emerge.

Spatial heterogeneity induces a marked increase in variability. Even when restricted to protocols belonging to the efficiency frontier under uniform conditions, the achieved gains span a wide range, including negative values. This indicates that, while the homogeneous frontier identifies high-performing protocols on average, their performance can vary substantially depending on the underlying spatial configuration.

Moreover, the effect of heterogeneity on treatment outcome is strongly regime-dependent. In normoxic conditions, the median gain remains approximately constant, reflecting the overall flatness of the response landscape and the limited sensitivity to protocol selection. In contrast, under hypoxic conditions, spatial heterogeneity leads to both larger potential improvements and substantially increased variability. In particular, for highly heterogeneous configurations (large Gini coefficient), the gain can decrease significantly and may even become negative, indicating that protocols selected under homogeneous assumptions can be compromised by unfavorable spatial arrangements.

An unbinned version of the same analysis, in which heterogeneous configurations are shown individually rather than pooled across Gini intervals, is reported in Supporting Figure S3. The same regime-dependent trend is recovered, indicating that binning is used only to improve visualization.

Overall, these results indicate that the efficiency frontier identified under uniform source assumptions remains qualitatively robust under spatial redistribution of oxygen, particularly in the hypoxic regime, where protocols with longer inter-fraction intervals, for sufficiently low heterogeneity, yield substantial gains. However, this robustness is primarily qualitative: spatial heterogeneity introduces significant variability in treatment outcomes, so that the performance of a given protocol can depend strongly on the specific geometric configuration. In normoxia, the frontier continues to provide limited benefit, in agreement with the uniform case, and spatial heterogeneity does not introduce a clear advantage in protocol selection. Rather, it mainly induces variability without altering the overall flatness of the response landscape.

These observations suggest that incorporating information on the spatial geometry of oxygen delivery could significantly improve protocol selection. In particular, geometry-aware selection may reduce the variability of TTP gains across heterogeneous configurations and, more importantly, may help exclude spatial regimes in which protocols selected under uniform assumptions yield negative gains relative to the SoC. This effect is especially relevant in hypoxic conditions, where large positive gains are possible but unfavorable geometries can compromise protocol performance. In normoxic conditions, spatial information may instead help identify the limited subset of configurations in which non-standard schedules provide a measurable advantage.

## Discussion

This study shows that spatial oxygen structure and phenotypic adaptation jointly alter radiotherapy protocol ranking. Rather than restricting the analysis to a limited number of predefined schedules under fixed radiosensitivity assumptions, we systematically explore the dose–interval plane and examine how protocol performance varies across oxygenation regimes and toxicity constraints. A summary of the main model-based findings and their implications is provided in Table 2.

**Table 2:** Key takeaways from the model analysis. The table summarizes how oxygenation regime, phenotypic adaptation, toxicity constraints, and oxygen-source geometry affect radiotherapy protocol ranking in the proposed framework

A first central result is that the effectiveness of a fractionation schedule is not intrinsic to the schedule itself, but depends critically on the TME. Under spatially uniform oxygen supply, we identify a region of near-optimal schedules—referred to as the therapeutic efficiency frontier—defined as the subset of dose–interval combinations that achieve near-maximal time-to-progression. In practice, this corresponds to the high-performance region of the dose–interval map obtained by systematically scanning fractionation protocols. This region exhibits a relatively regular structure, and deviations from standard-of-care protocols provide limited benefit in normoxic conditions. In contrast, under hypoxia the frontier changes shape and alternative schedules—particularly protracted or metronomic-like schemes—can substantially delay tumor regrowth. At the mechanistic level, this behavior is consistent with the interplay between the well-known reoxygenation effect following radiation delivery and phenotype redistribution (Steel et al. 1989). In particular, when reoxygenation compensates therapy-driven selection toward resistant traits, time-to-progression values remain comparable; when this compensation mechanism weakens, slower-proliferating hypoxia-adapted phenotypes alter the balance and reshape protocol performance.

The structure of the therapeutic efficiency frontier is further modulated by tissue tolerance. Changes in biologically effective dose and normal-tissue $(\alpha /\beta )_H$ ratio reshape the admissible region of the dose–interval plane. Under severe hypoxia, protocol selection becomes strongly governed by normal-tissue responsiveness. When tolerance is relatively high, hypofractionated schedules dominate the frontier, whereas decreasing tolerance induces a sharp transition toward hyperfractionated schemes. This finding aligns with the dynamics of slowly proliferating, late-responding tumors and is consistent with clinical observations suggesting that selected hypoxic tumors may benefit from hypofractionated strategies (Fowler et al. 2001).

The extension to intermediate oxygen supply levels clarifies that the benefit of schedule adaptation is non-monotone with respect to oxygen availability. Gains over standard-of-care were small under normoxia, increased in the intermediate hypoxic range, and decreased again under severe hypoxia. This indicates that moderate hypoxia represents a window in which oxygen is sufficiently low to reshape phenotypic selection and protocol ranking, but not so low as to globally suppress radiation efficacy. The barycenter trajectory of the efficiency frontier supports the same interpretation: as oxygenation decreases from normoxia to moderate hypoxia, the high-performance region shifts toward more protracted schedules; under severe hypoxia, it bends back toward larger fraction sizes and shorter intervals. Thus, the model does not simply predict that ’less oxygen’ uniformly favors alternative schedules. Instead, it identifies a specific oxygenation regime in which fractionation and timing have the largest effect.

A further result concerns the role of spatial geometry. In the heterogeneous-source analysis, we kept the total oxygen supply fixed and varied only its spatial organization. This construction separates the effect of oxygen-source geometry from the effect of total oxygen input. Protocols selected from the uniform-source efficiency frontier were then applied to heterogeneous configurations without re-optimization. The resulting gains show that the uniform-source frontier remains qualitatively informative, especially under hypoxia, where protracted schedules often continue to provide substantial benefit. At the same time, spatial heterogeneity introduces substantial outcome uncertainty, inducing large variability in treatment response. In highly polarized configurations, the gain may decrease substantially or even become negative. Therefore, spatial structure should not be interpreted as a small perturbation of an otherwise average oxygenation state. It can affect both the expected benefit and the reliability of a protocol selected under simplified assumptions. In this perspective, spatially resolved biomarkers of hypoxia, such as those obtained from functional imaging modalities (e.g. FMISO-PET), could provide more informative descriptors of the TME and support treatment selection beyond mean oxygenation levels (McNeal et al. 2024).

The model also provides a complementary perspective to standard LQ–OER formulations. Classical oxygen-dependent LQ models rescale radiosensitivity through oxygen availability but usually do not represent the phenotypic redistribution induced by hypoxic selection. Here, oxygen acts both directly, through radiosensitivity modulation, and indirectly, through the selection of phenotypes with different proliferative and radiotherapy-response traits. As discussed in the Supporting Information S5, this coupling allows the effective $\alpha /\beta$ behavior to change along the selected phenotypic trajectory, offering a possible mechanistic bridge between controlled oxygen-response experiments and clinical observations in which hypoxic or slow-growing tumors may benefit from hypofractionated treatment.

The present formulation relies on simplifying assumptions that enable a controlled investigation of treatment comparison under heterogeneous microenvironmental conditions. In particular, the spatial domain is one-dimensional, oxygen sources are prescribed rather than dynamically remodeled, and radiation-induced vascular remodeling is not explicitly represented (Cicchetti et al. 2020). These assumptions limit the quantitative interpretation of geometry-dependent variability, especially in realistic vascular networks. However, the main reranking mechanisms identified in this work do not arise solely from source geometry: protocol-dependent changes already emerge under spatially uniform oxygen supply, through the coupling between oxygenation, radiosensitivity, and phenotypic selection. The heterogeneous-source analysis should therefore be interpreted as a controlled perturbation of this baseline mechanism, showing that spatial organization can further modulate both the expected gain and the reliability of protocols selected under simplified assumptions. Extensions to higher-dimensional and dynamically evolving vascular geometries may refine the quantitative magnitude of these effects, but are not expected to remove the underlying dependence of protocol ranking on oxygenation and phenotypic adaptation.

Another simplifying assumption concerns tumor oxygen consumption, which is taken to be phenotype-independent. The present model does not explicitly resolve alternative metabolic pathways or phenotype-specific oxygen-consumption mechanisms. If, for example, hypoxia-adapted cells undergo metabolic reprogramming toward a more glycolytic phenotype and consequently consume less oxygen, their enrichment could reduce local oxygen depletion and promote reoxygenation. This additional feedback could modify the balance between hypoxic selection, proliferation, radiosensitivity, and treatment response, thereby shifting the quantitative predictions of the model. Other phenotype–metabolism relationships could lead to different feedbacks, and their impact would require an explicit phenotype-dependent description of oxygen uptake.

A further limitation concerns the representation of phenotypic adaptation following reoxygenation. The present model does not explicitly account for *hypoxic memory*, whereby cells may retain hypoxia-induced molecular and phenotypic programs for some time after reoxygenation (Rocha et al. 2021; Sadhu et al. 2026). The oxygen-dependent components of fitness and radiosensitivity at a given phenotype are determined by the current local oxygen level, and the local fitness optimum is correspondingly determined by this instantaneous oxygen environment (see Supporting Information S3). Although the effects of previous hypoxic exposure may persist indirectly through the evolving phenotypic composition of the tumor population, an explicit representation of hypoxic memory, as recently incorporated into a phenotype-structured PDE framework closely related to ours (Sadhu et al. 2026), could prolong hypoxia-associated behavior following reoxygenation and modify the quantitative balance between reoxygenation and hypoxia-driven selection that underlies treatment response and protocol ranking. This effect may be particularly relevant under the protracted, metronomic-like schedules favored under hypoxia. We regard this as a natural and promising model extension.

Building on this framework, future developments may incorporate more realistic vascular architectures, dynamic oxygen delivery, and patient-specific information derived from functional imaging or multi-omics profiling. The model also lends itself to optimal control formulations in which fractionation schedules are optimized under explicit tumor control and normal-tissue objectives, and may be integrated with dose-painting strategies targeting spatially resolved regions, such as resistant niches that remain a subject of active debate (Qiu et al. 2017).

A complementary extension concerns uncertainty quantification. In the present study, variability is introduced through Monte Carlo realizations of oxygen-source geometry, while phenotypic dynamics, radiosensitivity parameters, and treatment response are otherwise deterministic for a given configuration. Further stochastic extensions could explicitly model intrinsic variability in phenotypic evolution, radiosensitivity, and treatment response, allowing the robustness of treatment comparisons to be assessed under broader biological uncertainty.

Within these limitations, the proposed in silico approach offers a consistent quantitative setting for generating and testing hypotheses on how oxygenation, spatial heterogeneity, and phenotypic adaptation may affect radiotherapy schedule ranking. In particular, it provides a structured tool for exploring alternative fractionation strategies before considering more clinically constrained, patient-specific optimization settings.

## Supplementary Information

Below is the link to the electronic supplementary material.

Supplementary file 1 (pdf 967 KB)

## Data Availability

Not applicable.

## Code Availability

The code used to perform the simulations and generate the results presented in this study is available at https://staff.polito.it/marcello.delitala/code/submission_code.zip.
