# Supplementary material for: Oxygenation and Spatial Heterogeneity Shape Radiotherapy Protocol Ranking Through Phenotypic Adaptation
Source: Bull Math Biol. 2026 Oct 3;88(11):196. doi: 10.1007/s11538-026-01758-5 (PMC13633973; doi:10.1007/s11538-026-01758-5)
Supplement: Supplementary file 1 [file 11538_2026_1758_MOESM1_ESM.pdf]

Supporting Information for: Oxygenation and spatial heterogeneity shape radiotherapy protocol ranking through phenotypic adaptation

Francesco Albanese[1*], Giulia Chiari[2,3] and Marcello Edoardo Delitala[1]

[1*]Department of Mathematical Sciences "G. L. Lagrange", Politecnico di Torino, Torino, Italy.
[2]Basque Center for Applied Mathematics (BCAM), Bilbao, Spain.
[3]Mathematical Institute, University of Oxford, Oxford, United Kingdom.

*Corresponding author(s). E-mail(s): francesco_albanese@polito.it;

DOI: 10.1007/s11538-026-01758-5

This file contains supplementary material intended to support the results presented in the main text, including additional derivations, figures, and methodological details.

Throughout this Supporting Information, references to figures, equations, tables, and sections without the prefix "S" refer to the main text.

S1 Simulation details

S1.1 Spatial domain and tumor burden

Simulations are performed on the one-dimensional spatial domain $\Omega_s = [0, L_x]$ with $L_x = 2\,\text{cm}$. Recalling the volumetric interpretation of the cell density $n(t, x, u)$, the transverse dimensions are fixed to unit cross-sectional area ($1\,\text{cm}^2$) when computing the total tumor burden, so that

$$\Gamma(t) = \int_{\Omega_s} \int_{\Omega_p} n(t, x, u)\, du\, dx \tag{S1}$$

can be directly interpreted as the total number of tumor cells within the virtual tissue.

S1.2 Detection threshold and simulation endpoints

All simulations start at $t = 0$ and evolve until the tumor reaches a prescribed detection threshold. Following Erdi (2012), clinical detectability is typically associated with a minimal tumor spheroid of radius $r = 0.35$ cm. We convert this detectable volume into a total cell number by assuming an effective mean cellular packing equal to 70% of the reference carrying capacity. This gives the detectability threshold

$$\Gamma_{\mathrm{RT}} = 1.3 \cdot 10^8 \text{ cells.} \tag{S2}$$

Radiotherapy is initiated when the total tumor burden exceeds this threshold:

$$t_0^{\mathrm{RT}} = \inf \{t \ : \ \Gamma(t) \geq \Gamma_{\mathrm{RT}}\}, \tag{S3}$$

and numerical simulations end at relapse time

$$t_{\mathrm{end}} = \inf \left\{t > t_0^{\mathrm{RT}} \ : \ \Gamma(t) \geq \Gamma_{\mathrm{RT}}\right\}. \tag{S4}$$

S1.3 Parameter estimates

A large subset of the model parameters is informed by estimates available in the empirical literature. Accordingly, the adopted parameter configuration is intended to capture generic tumor behavior. When tumor-specific data are available, the model may be further calibrated to reflect the characteristics of a particular cancer type. The complete set of parameter values is reported in Table 1. Whenever additional detail on parameter derivation or calibration is needed, the reader is referred to the relevant equations or to the sections where a full derivation is provided.

S1.4 Initial and boundary conditions

At time $t = 0$, the tumor cell density is prescribed as a spatially localized distribution with phenotypes concentrated near the radiation-sensitive state:

$$n(0, x, u) = n_0(x, u) := A \exp\left(-\frac{(x - x_0)^2}{2\sigma_x^2}\right) \exp\left(-\frac{u^2}{2\sigma_u^2}\right), \qquad (x, u) \in \Omega_s \times \Omega_p, \tag{S5}$$

where $x_0 = L_x/2$ denotes the initial tumor location, while $\sigma_x > 0$ and $\sigma_u > 0$ control the spatial and phenotypic spread, respectively. The amplitude $A = 10^5\,\mathrm{cell/cm^3}$ is chosen so that the initial tumor density lies well below the prescribed carrying-capacity scale K throughout the spatial domain. This ensures that the system initially evolves in a low-density regime, before density-dependent crowding becomes relevant. The calibration of the crowding coefficient κ based on K is described in Section S4.

The initial oxygen concentration is prescribed through a consistent initial profile. Since oxygen dynamics evolve on a timescale that is several orders of magnitude faster than tumor cell proliferation, the specific choice of the initial condition has a negligible impact on the subsequent dynamics.

No-flux boundary conditions are imposed both in space and in phenotype for the tumor cell density, and in space for the oxygen field. Biologically, these conditions

represent physical and functional barriers that prevent cell escape from the considered tissue portion and exclude phenotypic flux across the extreme resistance states, while assuming no external oxygen exchange across the tissue boundaries.

S1.5 Numerical methods

The spatio–phenotypic system for the tumor cell density $n(t, x, u)$ and the oxygen field $O(t, x)$, defined in Eqs. (1)-(9), is solved numerically by finite differences on uniform grids in space and phenotype. Diffusion operators are approximated by second-order centered finite differences. Time integration is performed using a first-order explicit (forward Euler) scheme combined with operator splitting of reaction, phenotypic diffusion, and spatial diffusion terms. The oxygen equation is advanced by an explicit reaction-diffusion update, with internal substepping when required by stability constraints. Radiotherapy is implemented in an event-driven fashion: the time step is adaptively adjusted to land exactly on scheduled dosing times, at which the active cell population is instantaneously updated according to the modified linear-quadratic survival model proposed in Eq. (5).

S1.6 Computational details

All simulations were implemented in MATLAB R2024b. The spatial and phenotypic domains were discretized using uniform grids with mesh sizes $\Delta x = 2\,\text{cm}/100$ and $\Delta u = 1/100$, respectively. One ghost point was included at each boundary to impose no-flux boundary conditions. The oxygen field was discretized on the same spatial grid. Integral quantities were computed using native trapezoidal-rule routines.

Given the large number of independent model evaluations required by the dose–interval scans and by the Monte Carlo exploration of heterogeneous oxygen-source configurations, simulations were parallelized at the level of independent runs. Each dose–interval map was obtained from $50 \times 50 = 2500$ independent simulations, corresponding to the explored combinations of single-fraction dose and inter-fraction interval. In the heterogeneous-source analysis, each set of 2500 protocols was evaluated over 100 independently generated oxygen-source configurations, yielding 2500×100 simulations for each oxygenation regime considered.

Large batches of simulations were executed on a high-performance computing cluster using the SLURM workload manager. This parallelization did not alter the numerical scheme described above: each job corresponded to an independent realization of a prescribed protocol and oxygen-source configuration. The computational cost varied across regimes, mainly because oxygenation level, spatial heterogeneity, and treatment response affect the simulated time-to-progression and therefore the number of time steps required before relapse. A typical single simulation required approximately 2 minutes in normoxic or weakly heterogeneous settings, and up to approximately 10 minutes in hypoxic and/or highly heterogeneous configurations. Thus, the exploration of dose–interval protocols and oxygen-source geometries was computationally intensive but naturally suited to parallelization, since individual simulations were independent.

S2 Indicator definitions

S2.1 Biologically Effective Dose (BED)

The *biologically effective dose* (BED) associated with a fractionation protocol $\mathcal{P} = (d, \Delta, N_f)$ is defined, according to the linear–quadratic formulation and its application to fractionated radiotherapy (Fowler 1989), as

$$\mathrm{BED}(\mathcal{P}) = N_f\, d \left(1 + \frac{d}{(\alpha/\beta)_H}\right), \tag{S6}$$

where N_f is the number of fractions and d the dose per fraction (Gy). The $(\alpha/\beta)_H$ ratio characterizes the fractionation sensitivity of the surrounding healthy tissue, with larger values associated with early-responding tissues and smaller values with late-responding tissues (Freedman et al. 2013). The BED provides a scalar measure of the expected biological effect of a fractionation schedule and is here used to quantify normal-tissue toxicity, enabling comparison across protocols under a common constraint.

S2.2 Mean phenotype

To quantify tumor adaptation to therapy and to microenvironmental stress induced by hypoxia, the *mean phenotype* is introduced:

$$\bar{u}(t) = \frac{\displaystyle\int_{\Omega_p}\int_{\Omega_s} u\, n(t,x,u)\,\mathrm{d}x\,\mathrm{d}u}{\displaystyle\int_{\Omega_s} \rho(t,x)\,\mathrm{d}x}. \tag{S7}$$

This quantity represents the average resistance level of the tumor population at time t, accounting for both spatial structure and phenotypic heterogeneity. This and similar averaged phenotypic indicators have been widely used in phenotypically structured models to characterize the macroscopic evolutionary dynamics of heterogeneous populations; see, for instance, Lorenzi et al. (2025) and references therein.

S2.3 Time-to-progression (TTP)

The performance of a treatment protocol is quantified through the *time-to-progression* (TTP), which serves as the primary efficacy indicator in our simulations. TTP measures the time interval between treatment initiation and tumor relapse:

$$\mathrm{TTP} := t_{\mathrm{end}} - t_0^{\mathrm{RT}}. \tag{S8}$$

Such metrics have been used in previous studies to assess and compare treatment strategies (see, e.g., Henares-Molina et al. 2017; Brüningk et al. 2021).

S3 Existence and uniqueness of the fittest phenotype

For each spatial location $x \in \Omega_s$ and time $t \in [0, t_{\text{end}}]$, the local oxygen concentration $O(t, x)$ induces a fitness landscape over the phenotypic trait $u \in \Omega_p = [0, 1]$ through the reaction term $R(t, x, u)$ in Eq. (2). Fixing (t, x), we can regard the reaction term as a function of u only and write

$$R(u; t, x) = r_0 + \gamma_O\, M\big(O(t,x)\big)\,\big(1-u^2\big) - \gamma_H\big(1 - M\big(O(t,x)\big)\big)\,\big(1-u\big)^2 - \kappa\,\rho(t,x), \quad \text{(S9)}$$

where $M(\cdot)$ is the Hill-type function introduced in Eq. (3) and $\rho(t, x)$ is the local total cell density defined in Eq. (4). Since the crowding term $-\kappa\,\rho(t, x)$ does not depend on u, the phenotype that maximizes $R(u; t, x)$ coincides with the maximizer of

$$\widetilde{R}(u; t, x) = r_0 + \gamma_O\, M\big(O(t,x)\big)\,\big(1-u^2\big) - \gamma_H\big(1 - M\big(O(t,x)\big)\big)\,\big(1-u\big)^2. \quad \text{(S10)}$$

For notational brevity, in what follows we denote $M = M\big(O(t, x)\big)$ and omit the explicit dependence on (t, x). The first derivative of $\widetilde{R}$ with respect to u is

$$\frac{d}{du}\widetilde{R}(u) = -2\,\gamma_O\, M\, u + 2\,\gamma_H\,(1-M)\,(1-u). \quad \text{(S11)}$$

The critical point u^* is obtained by solving $\frac{d}{du}\widetilde{R}(u) = 0$, which yields

$$u^* = \frac{\gamma_H(1-M)}{\gamma_O M + \gamma_H(1-M)}. \quad \text{(S12)}$$

Using the assumptions $\gamma_O > 0$, $\gamma_H > 0$ and $M \in [0, 1]$, it follows that $0 \le \gamma_H(1-M) \le \gamma_O M + \gamma_H(1-M)$, so that $u^* \in [0, 1]$. Therefore, u^* is an admissible phenotype in Ω_p. To show that u^* is the unique global maximizer of $R(u; t, x)$ with respect to u, we compute the second derivative:

$$\frac{d^2}{du^2}\widetilde{R}(u) = -2\big(\gamma_O M + \gamma_H(1-M)\big) < 0 \quad \text{for all } u \in [0,1], \quad \text{(S13)}$$

which confirms that $\widetilde{R}(u)$ (and hence $R(u; t, x)$) is strictly concave in u. As a consequence, u^* defined in (S12) is the unique fittest phenotype at position x and time t. The dependence of u^* on the oxygen concentration $O(t, x)$ is encoded through the function $M\big(O(t, x)\big)$, which is strictly increasing in O. In particular,

$$O \to 0 \;\Rightarrow\; M(O) \to 0 \;\Rightarrow\; u^* \to 1, \qquad O \to +\infty \;\Rightarrow\; M(O) \to 1 \;\Rightarrow\; u^* \to 0. \quad \text{(S14)}$$

Therefore, in highly hypoxic regions the selected phenotype tends toward $u^* \approx 1$, i.e. toward highly resistant traits, whereas in well-oxygenated regions it tends toward $u^* \approx 0$, corresponding to more proliferative and less resistant phenotypes. This behavior is

consistent with the modeling interpretation of u adopted in the main text and with the eco-evolutionary role of hypoxia in selecting resistant tumor cell clones.

S4 Calibration of the crowding coefficient

For fixed (t, x, u), the net proliferation rate can be written as

$$R(t, x, u) = F\big(O(t, x), u\big) - \kappa\rho(t, x), \tag{S15}$$

where

$$F(O, u) = r_0 + \gamma_O M(O)(1 - u^2) - \gamma_H\big(1 - M(O)\big)(1 - u)^2. \tag{S16}$$

Since $M(O) \in [0, 1)$ and $u \in [0, 1]$, the intrinsic growth term satisfies

$$\sup_{O,u} F(O, u) = r_0 + \gamma_O. \tag{S17}$$

We calibrate κ so that, when the local density reaches K, even the most favorable growth conditions do not yield positive net proliferation:

$$r_0 + \gamma_O - \kappa K = 0. \tag{S18}$$

Accordingly,

$$\kappa = \frac{r_0 + \gamma_O}{K}. \tag{S19}$$

In this sense, K is interpreted as an upper-bound carrying capacity. Throughout the model, the cell density $n(t, x, u)$ is interpreted as volumetric (cells/cm^3). We set $K = 10^9$ cells/cm^3, corresponding to the typical number of cells in a $1\,\text{cm}^3$ tumor (Del Monte 2009).

S5 An integrated perspective on cellular radiosensitivity

In the LQ framework, the parameters α and β quantify cell death from single- and multiple-track events (McMahon 2018). Reported values show substantial variability across tumor types (van Leeuwen et al. 2018), which is typically larger than the ranges assumed in modeling studies. The ratio α/β represents the dose at which linear and quadratic contributions are equal and is therefore relevant for fractionation sensitivity.

Oxygen effects are commonly incorporated through the Oxygen Enhancement Ratio (OER), defined as the factor by which radiation dose must be increased under hypoxia to achieve the same biological effect as in normoxia. This leads to the rescaling

$$\alpha_{\text{norm}} = \text{OER}\,\alpha_{\text{hyp}}, \quad \beta_{\text{norm}} = \text{OER}^2\,\beta_{\text{hyp}}, \tag{S20}$$

implying $\alpha_{\text{hyp}}/\beta_{\text{hyp}} = \text{OER}\,\alpha_{\text{norm}}/\beta_{\text{norm}}$. Typical values of OER range up to approximately 2.5–3 depending on oxygenation level. However, experimental evidence is not fully consistent with this prediction. While in vitro studies support the OER-based

scaling (Stea and Gordon 2012), clinical observations often report lower α/β ratios in hypoxic tumors, which are frequently treated with hypofractionated schedules (Wang et al. 2003; Mohamed et al. 2023; Ritter et al. 2009). In addition, hypoxia affects DNA repair and promotes resistant, stem-like phenotypes. To account for these effects, we define α and β as functions of oxygen concentration and phenotype in Eq. (6). Starting from baseline values $\tilde{\alpha}$ and $\tilde{\beta}$, we introduce phenotype-dependent amplitudes Δ_α and Δ_β, combined with an oxygen-dependent rescaling consistent with the OER formulation. Parameters are chosen so that, for $\mathrm{OER}_{\max} = 3$, the resulting values of α and β span the ranges reported in the literature (van Leeuwen et al. 2018).

In this setting, hypoxia directly reduces radiosensitivity through the standard OER mechanism, while hypoxia-driven selection shifts the population toward more resistant phenotypes. The combined effect of oxygenation and phenotypic composition modifies both radiosensitivity and the α/β ratio. At fixed phenotype, decreasing oxygen increases α/β, as predicted by the classical formulation. Along the phenotype selected by the system dynamics (see Supporting Information S3), the ratio instead decreases toward hypoxia due to the concurrent shift toward resistant traits (Fig. S1). This formulation provides a unified description that captures both controlled conditions, where oxygen and phenotype can be varied independently (*in vitro*), and evolving populations subject to selection (*in vivo*).

S6 Oxygen dynamics quantification: baseline consumption and tumor uptake

This section details the derivation and calibration of the oxygen decay and consumption parameters used in Eq. (9). The goal is to relate the effective coefficients λ_O and ζ_O to physiological quantities reported in the literature. To parameterize the baseline oxygen decay term $-\lambda_O O$, we relate it to the physiological oxygen consumption of healthy tissue. Assuming homeostatic conditions, the density of healthy cells is fixed at the carrying capacity $K_H = 5 \cdot 10^8\,\mathrm{cells/cm^3}$. We denote by $\tilde{\zeta}_H$ the per-cell oxygen consumption rate, $\tilde{\zeta}_H = 5 \cdot 10^{-18}\,\mathrm{mol\,cell^{-1}\,s^{-1}} = 4.32 \cdot 10^{-13}\,\mathrm{mol\,cell^{-1}\,day^{-1}}$, within the range reported in Wagner et al. (2011). Using the molar volume of oxygen $\eta_O = 2.24 \cdot 10^4\,\mathrm{ml\,O_2/mol}$ and a reference normoxic level $O_{\mathrm{ref}} = 2.16 \cdot 10^{-3}\,\mathrm{ml\,O_2/cm^3}$, we define the volumetric consumption coefficient as $\zeta_H := \tilde{\zeta}_H \eta_O / O_{\mathrm{ref}}$, which yields

$$\lambda_O = \zeta_H K_H. \tag{S21}$$

This corresponds to a linearization of volumetric oxygen consumption around the reference normoxic state. The above quantities are expressed in the units adopted in the referenced study. The resulting parameter is defined consistently with the model formulation, and its final value is reported in Table 1.

Oxygen consumption by tumor cells is modeled through the nonlinear term $-\zeta_O \rho O$, where ρ denotes the tumor cell density. The consumption rate is assumed phenotype-independent. Neglecting diffusion, one may write $\partial_t O = V_O(x) - \zeta_H K_H O - \zeta_T \rho O$, where ζ_T is the tumor per-cell consumption coefficient. Since the term $-\lambda_O O$ already accounts for healthy tissue consumption, the tumor contribution is interpreted as an

excess uptake due to the replacement of healthy cells. Assuming a higher compressibility of tumor cells, allowing for up to a one-to-two replacement ratio, we obtain the effective formulation

$$\partial_t O = V_O(x) - \lambda_O O - \zeta_O \rho O, \tag{S22}$$

with $\zeta_O = \zeta_T - \zeta_H/2$. Accordingly, we adopt a per-cell tumor consumption rate $\tilde{\zeta}_T = 6.7 \cdot 10^{-17}\,\mathrm{mol\,cell^{-1}\,s^{-1}} = 5.79 \cdot 10^{-12}\,\mathrm{mol\,cell^{-1}\,day^{-1}}$, within the range reported in Wagner et al. (2011), yielding the coefficient ζ_O listed in Table 1.

S7 Quantification of the therapeutic efficiency frontier

We consider the set of fractionated radiotherapy protocols used to construct the dose–interval maps. Each protocol consists of identical dose fractions delivered at a constant inter-fraction interval, with the number of fractions determined by the imposed BED constraint. For each $i \in \mathcal{I}$, where $\mathcal{I}$ denotes the index set of all protocols explored, we denote by $\mathcal{P}_i$ the i-th simulated protocol. The corresponding dose–interval pair is (d_i, Δ_i), and TTP_i denotes the time-to-progression obtained from the simulation. We define

$$\mathrm{TTP}_{\max} := \max_{i \in \mathcal{I}} \mathrm{TTP}_i \tag{S23}$$

as the maximum time-to-progression attained in that protocol set. The *therapeutic efficiency frontier* is then defined as the set of protocols

$$\mathcal{F} := \{\, \mathcal{P}_i \;:\; i \in \mathcal{I},\; \mathrm{TTP}_i \geq 0.8 \cdot \mathrm{TTP}_{\max} \,\}. \tag{S24}$$

Thus, $\mathcal{F}$ identifies the simulated protocols achieving at least 80% of the maximum predicted TTP. It can therefore be interpreted as the set of near-optimal protocols with respect to this performance metric.

To provide a compact summary of the typical location of the therapeutic efficiency frontier in the (d, Δ) plane, we introduce its TTP-*weighted barycenter*. Specifically, the barycenter $(\bar{d}, \bar{\Delta})$ is defined as

$$\bar{d} := \frac{\sum_{\mathcal{P}_i \in \mathcal{F}} d_i \, \mathrm{TTP}_i}{\sum_{\mathcal{P}_i \in \mathcal{F}} \mathrm{TTP}_i}, \qquad \bar{\Delta} := \frac{\sum_{\mathcal{P}_i \in \mathcal{F}} \Delta_i \, \mathrm{TTP}_i}{\sum_{\mathcal{P}_i \in \mathcal{F}} \mathrm{TTP}_i}. \tag{S25}$$

By construction, protocols yielding larger TTP values contribute more strongly to the location of the barycenter. The barycenter is not intended to identify an optimal treatment schedule. Rather, it provides a low-dimensional descriptor of the region in the dose–interval space associated with highly effective regimens. This representation facilitates comparisons across different oxygenation conditions and toxicity constraints, and is therefore used throughout the manuscript to track qualitative shifts in the therapeutic efficiency frontier.

S8 Construction of heterogeneous oxygen sources

S8.1 Monte Carlo construction of oxygen sources

To investigate the effect of spatial heterogeneity in oxygen delivery, we construct an ensemble of oxygen source configurations with identical total input and varying spatial organization. The ensemble is generated through a Monte Carlo procedure, so that each realization corresponds to a different spatial redistribution of the same total oxygen supply. In the spatially uniform case, the oxygen source is defined as

$$V_O(x) = I_0\,\lambda_O, \qquad x \in \Omega_s = [0, L_x], \tag{S26}$$

which yields a constant oxygen supply across the domain. In the heterogeneous setting, for each Monte Carlo realization $m = 1, \dots, M$, we construct a raw oxygen source profile as a superposition of n_V localized Gaussian-shaped contributions:

$$V_{\mathrm{raw}}^{(m)}(x) = \sum_{i=1}^{n_V} w_i^{(m)} \exp\left(-\frac{\left(x - c_i^{(m)}\right)^2}{2\left(\sigma_i^{(m)}\right)^2} \right), \qquad x \in \Omega_s. \tag{S27}$$

The parameter n_V controls the number of localized oxygen-releasing regions and therefore the spatial granularity of oxygen delivery. It should not be interpreted as the literal number of physical vessels, but rather as a modeling parameter determining the complexity of the oxygen source. For each realization, the centers $c_i^{(m)}$ are sampled independently and uniformly over the spatial domain,

$$c_i^{(m)} \sim \mathcal{U}[0, L_x], \tag{S28}$$

while the widths $\sigma_i^{(m)}$ are sampled independently from a uniform distribution over a prescribed range. These widths do not have a direct biological interpretation and are introduced as a modeling choice to generate a broad variety of spatial configurations. The relative amplitudes $w_i^{(m)}$ are sampled independently from a uniform distribution. Since the raw profile $V_{\mathrm{raw}}^{(m)}$ generally has a realization-dependent integral, we apply a realization-dependent scaling factor to enforce conservation of the total oxygen supply:

$$V_O^{(m)}(x) = \eta^{(m)}\, V_{\mathrm{raw}}^{(m)}(x), \tag{S29}$$

where

$$\eta^{(m)} = \frac{I_0\, L_x\, \lambda_O}{\displaystyle\int_{\Omega_s} V_{\mathrm{raw}}^{(m)}(x)\, dx}. \tag{S30}$$

With this normalization, all realizations satisfy

$$\int_{\Omega_s} V_O^{(m)}(x)\, dx = I_0\, L_x\, \lambda_O, \tag{S31}$$

so that they share the same total oxygen supply and differ only in the spatial geometry of delivery. This construction allows us to isolate the effect of spatial heterogeneity on treatment outcomes by comparing different geometric configurations under identical global oxygen supply.

S8.2 Quantification of spatial heterogeneity

To provide a compact descriptor of spatial heterogeneity, we associate to each oxygen-source realization m a normalized Gini coefficient $\mathcal{G}^{(m)}$, computed from the corresponding discretized source profile $V_O^{(m)}(x)$. Let

$$v^{(m)} = \left(v_1^{(m)}, \ldots, v_n^{(m)}\right) \tag{S32}$$

denote the values of $V_O^{(m)}(x)$ on the spatial grid, normalized so that

$$\sum_{k=1}^{n} v_k^{(m)} = 1. \tag{S33}$$

Here, $v_k^{(m)}$ denotes the value at the k-th spatial grid point. Let

$$v_{(1)}^{(m)} \leq v_{(2)}^{(m)} \leq \cdots \leq v_{(n)}^{(m)} \tag{S34}$$

denote the same values sorted in non-decreasing order. The normalized Gini coefficient of realization m is then defined as

$$\mathcal{G}^{(m)} = \frac{1}{n-1} \sum_{k=1}^{n} (2k - n - 1)\, v_{(k)}^{(m)}. \tag{S35}$$

The normalization by $n - 1$ ensures that $\mathcal{G}^{(m)} \in [0, 1]$ on the finite spatial grid. By construction, $\mathcal{G}^{(m)} = 0$ corresponds to a perfectly uniform oxygen source, whereas $\mathcal{G}^{(m)} = 1$ corresponds to the limiting case in which all oxygen supply is concentrated at a single grid point. Therefore, the Gini coefficient primarily captures the degree of *polarization* of the oxygen source, i.e., how strongly oxygen delivery is concentrated in localized regions. In this work, it is adopted as a first, compact descriptor of spatial heterogeneity. Other indicators could in principle be used to characterize different aspects of the spatial organization of the oxygen distribution.

S9 Schedule-domain robustness analysis

The work presented in Henares-Molina et al. (2017) represents a key contribution to the study of optimal radiotherapy protocols while also raising important open questions. Our work builds on these aspects by further investigating how the evolutionary and environmental features of the tumor influence the selection of optimal treatment

strategies. We have shown that oxygenation and tumor heterogeneity strongly affect the benefit of alternative protocols over the standard-of-care.

We further examine robustness with respect to the explored schedule domain by repeating the dose–interval scans under progressively restricted admissible inter-fraction interval ranges, while keeping the BED constraint unchanged. We test whether the main oxygenation-dependent patterns reported in the manuscript—including the emergence of larger gains away from SoC in intermediate hypoxia—persist when the maximum allowable spacing between fractions is reduced. Specifically, we considered

$$\Delta_{\max} \in \{7, 14, 30, 60, 100\} \text{ days} \tag{S36}$$

where $\Delta_{\max} = 100$ corresponds to the extended range used in the main-text dose–interval maps. Here, I_0 denotes the reference tumor-free oxygenation level used to set the total oxygen supply. For each pair $(I_0, \Delta_{\max})$, let $\mathcal{I}(I_0; \Delta_{\max})$ denote the index set of all simulated protocols in the restricted scan, with $\mathcal{P}_i$ denoting the i-th simulated protocol. The restricted scan contains only protocols satisfying the same BED constraint used in the main text and the additional condition $\Delta_i \leq \Delta_{\max}$. For each restricted scan, we first define the maximum TTP attained over the admissible protocol set as

$$\mathrm{TTP}_{\max}(I_0; \Delta_{\max}) := \max_{i \in \mathcal{I}(I_0; \Delta_{\max})} \mathrm{TTP}_i(I_0; \Delta_{\max}) \tag{S37}$$

The corresponding therapeutic efficiency frontier is then defined as

$$\mathcal{F}(I_0; \Delta_{\max}) := \{\mathcal{P}_i : i \in \mathcal{I}(I_0; \Delta_{\max}),\ \mathrm{TTP}_i(I_0; \Delta_{\max}) \geq 0.8 \cdot \mathrm{TTP}_{\max}(I_0; \Delta_{\max})\} \tag{S38}$$

This definition follows Section S7, with the admissible set now restricted by $\Delta_i \leq \Delta_{\max}$.

For each protocol in this frontier, the gain over standard-of-care is computed by comparing its TTP with the SoC TTP under the same oxygenation level. We therefore define the median gain as

$$\widetilde{G}(I_0; \Delta_{\max}) := \mathrm{median}_{\mathcal{P}_i \in \mathcal{F}(I_0; \Delta_{\max})} \left(\mathrm{TTP}_i(I_0; \Delta_{\max}) - \mathrm{TTP}_{\mathrm{SoC}}(I_0)\right) \tag{S39}$$

where $\mathrm{TTP}_{\mathrm{SoC}}(I_0)$ denotes the TTP obtained with the standard-of-care schedule at the same oxygenation level. Since the SoC schedule has $\Delta_{\mathrm{SoC}} = 1$ day, it remains admissible for all restricted domains considered here. Hence, the SoC baseline depends on I_0, but not on $\Delta_{\max}$. We also compute the median TTP over the same frontier,

$$\widetilde{\mathrm{TTP}}(I_0; \Delta_{\max}) := \mathrm{median}_{\mathcal{P}_i \in \mathcal{F}(I_0; \Delta_{\max})} \mathrm{TTP}_i(I_0; \Delta_{\max}) \tag{S40}$$

which provides the typical performance of the near-optimal protocols. In addition, we compute the TTP-weighted frontier barycenter $(\bar{d}, \bar{\Delta})$ as in Section S7. Finally, to compare relative improvements across oxygenation levels with different SoC baselines, we also report the median gain normalized by $\mathrm{TTP}_{\mathrm{SoC}}(I_0)$.

Fig. S2 summarizes these complementary aspects of the restricted-frontier analysis.

The top-left panel reports the median gain over SoC across protocols in $\mathcal{F}(I_0; \Delta_{\max})$. The gain depends non-monotonically on oxygenation, with the largest improvements in the intermediate hypoxic range. Restricting $\Delta_{\max}$ reduces the attainable median gains, especially for short admissible intervals, while preserving the same qualitative oxygenation-dependent trend.

The top-right panel reports the TTP-weighted barycenters $(\bar{d}, \bar{\Delta})$ of the corresponding frontiers. These barycenters identify the typical location of the near-optimal region in the dose–interval plane. At intermediate oxygenation, the frontier shifts toward lower per-fraction doses and longer inter-fraction intervals. As $\Delta_{\max}$ decreases, this shift is progressively constrained by the imposed upper bound on the admissible spacing between fractions. Because the frontier is recomputed within each restricted schedule domain, this constraint affects both components of the barycenter: in addition to reducing $\bar{\Delta}$, it also tends to move the near-optimal region toward lower values of $\bar{d}$, particularly in the intermediate hypoxic range.

The bottom-left panel reports the median TTP of protocols in $\mathcal{F}(I_0; \Delta_{\max})$, together with the corresponding SoC baseline. The median TTP of near-optimal schedules peaks at intermediate oxygenation, whereas near normoxia it approaches the SoC baseline, consistently with the flatter dose–interval landscape observed in the main text.

The bottom-right panel reports the same median gain shown in the top-left panel after normalization by the corresponding SoC baseline. This representation shows that the proportional benefit of schedule adaptation is maximal at intermediate hypoxia and becomes modest near normoxia and under severe hypoxia.

Overall, these results show that the qualitative conclusions reported in the main text are robust to the choice of the explored inter-fraction interval range. The same oxygenation-dependent patterns persist under progressively restricted $\Delta_{\max}$, although the attainable magnitude of the gain and the accessible region of the dose–interval plane are reduced when the maximum allowed spacing between fractions is shortened.

S10 Hill-coefficient sensitivity analysis

The Hill-type function introduced in Eq. (3) modulates the oxygen-dependent proliferative contribution and can be written more generally as

$$M(O; n_H) = \frac{O^{n_H}}{O^{n_H} + \alpha_O^{n_H}}, \tag{S41}$$

where $n_H > 0$ controls the steepness of the transition between low- and high-oxygen conditions. In the main analysis, we set $n_H = 4$ as a modeling choice, providing a sufficiently sharp transition and an effectively saturated response within the normoxic range.

Changing n_H modifies the sharpness of the oxygen response without altering either its limiting values or its half-saturation point, since

$$M(\alpha_O; n_H) = \frac{1}{2} \tag{S42}$$

for every n_H. The Hill coefficient therefore determines how rapidly the response approaches its low- and high-oxygen plateaus while leaving the characteristic transition oxygen level unchanged.

This distinction is particularly relevant for small Hill coefficients. At the reference normoxic oxygen level $O_M = 72.2$ mmHg, the response is approximately

$$M(O_M; 1) = 0.826, \qquad M(O_M; 2) = 0.958, \qquad M(O_M; 3) = 0.991, \tag{S43}$$

whereas $M(O_M; n_H) > 0.998$ for $n_H \geq 4$. Thus, $n_H = 1$, and to a lesser extent $n_H = 2$, produces a substantially more gradual transition and does not reproduce the same effective saturation of the oxygen-dependent proliferative contribution under normoxic conditions. Conversely, $n_H \geq 3$ yields an oxygen response that is already effectively saturated within the normoxic range while still spanning different transition steepnesses around $O = \alpha_O$.

To determine whether the treatment-selection results depend on this modeling choice, we repeated the complete dose–interval analysis underlying the main-text treatment landscapes for

$$n_H \in \{1, 2, 3, 4, 5, 6\}, \tag{S44}$$

under both the normoxic condition $I_0 = O_M$ and the hypoxic condition $I_0 = O_m$. The analysis was performed in the spatially uniform oxygen-source setting used for main-text Fig. 1; spatial heterogeneity in the oxygen source was not considered here. All remaining model parameters, simulation settings, the BED constraint, and the dose–interval protocol domain were kept unchanged.

Following the definitions introduced in Section S7, for each Hill coefficient and oxygenation condition we independently define the corresponding *therapeutic efficiency frontier* as

$$\mathcal{F}_{n_H}(I_0) = \left\{\mathcal{P} : \mathrm{TTP}_{n_H}(\mathcal{P}; I_0) \geq 0.8 \cdot \mathrm{TTP}^{\max}_{n_H}(I_0)\right\}, \tag{S45}$$

where $\mathcal{P} = (d, \Delta)$ denotes a treatment protocol in the dose–interval domain and $\mathrm{TTP}^{\max}_{n_H}(I_0)$ denotes the maximum TTP obtained over that domain for the corresponding value of n_H and oxygenation condition. The frontier therefore identifies the set of near-optimal treatment protocols for each value of n_H and oxygenation condition. In the following, the explicit dependence on I_0 is omitted for notational simplicity, since all comparisons are performed separately for each oxygenation condition.

The characteristic location of each therapeutic efficiency frontier is summarized through its *TTP-weighted barycenter*, defined as

$$\bar{d}_{n_H} = \frac{\displaystyle\sum_{\mathcal{P}\in\mathcal{F}_{n_H}} d_{\mathcal{P}}\, \mathrm{TTP}_{n_H}(\mathcal{P})}{\displaystyle\sum_{\mathcal{P}\in\mathcal{F}_{n_H}} \mathrm{TTP}_{n_H}(\mathcal{P})}, \qquad \bar{\Delta}_{n_H} = \frac{\displaystyle\sum_{\mathcal{P}\in\mathcal{F}_{n_H}} \Delta_{\mathcal{P}}\, \mathrm{TTP}_{n_H}(\mathcal{P})}{\displaystyle\sum_{\mathcal{P}\in\mathcal{F}_{n_H}} \mathrm{TTP}_{n_H}(\mathcal{P})}. \tag{S46}$$

The barycenter identifies the characteristic location of the near-optimal protocols in the dose–interval plane, weighted by their TTP values.

We then compare each alternative Hill coefficient with the reference case $n_H = 4$ using three complementary measures. First, we quantify the geometrical similarity

between the corresponding therapeutic efficiency frontiers through the Jaccard index,

$$J_{n_H} = \frac{|\mathcal{F}_{n_H} \cap \mathcal{F}_4|}{|\mathcal{F}_{n_H} \cup \mathcal{F}_4|}. \tag{S47}$$

Here, the numerator counts the protocols that belong simultaneously to the frontier obtained for n_H and to the reference frontier $\mathcal{F}_4$, whereas the denominator counts all protocols belonging to at least one of the two frontiers. Thus, J_{n_H} measures the fraction of the combined near-optimal protocol set that is shared by the two cases. A value of $J_{n_H} = 1$ corresponds to identical frontiers, while $J_{n_H} = 0$ corresponds to non-overlapping frontiers. In Fig. S4, the Jaccard index is reported as a percentage for readability; larger percentages therefore indicate greater geometrical agreement with the reference case.

Second, changes in the characteristic protocol location are quantified through the barycenter shifts

$$\delta d_{n_H} = \bar{d}_{n_H} - \bar{d}_4, \qquad \delta\Delta_{n_H} = \bar{\Delta}_{n_H} - \bar{\Delta}_4. \tag{S48}$$

These quantities measure whether changes in the Hill coefficient displace the high-performing region toward systematically different dose or inter-fraction interval regimes.

Finally, we quantify how the predicted TTP changes when the Hill coefficient is varied while keeping the treatment protocol fixed. Let $\mathcal{D}$ denote the complete dose–interval protocol domain. As detailed in Section S1.6, $\mathcal{D}$ consists of a 50×50 grid of dose–interval combinations, corresponding to 2500 treatment protocols simulated independently. For each $\mathcal{P} \in \mathcal{D}$, we compare the TTP obtained with n_H with the corresponding value obtained for the reference case $n_H = 4$, and summarize these differences through

$$\mathrm{RMSE}_{n_H} = \sqrt{\frac{1}{|\mathcal{D}|} \sum_{\mathcal{P} \in \mathcal{D}} \left[\mathrm{TTP}_{n_H}(\mathcal{P}) - \mathrm{TTP}_4(\mathcal{P})\right]^2}. \tag{S49}$$

The RMSE is expressed in years and measures the typical TTP deviation from the reference $n_H = 4$ case over the complete protocol domain.

The results are summarized in Fig. S4. A clear separation emerges between $n_H = 1, 2$, which produce the largest deviations, and the effectively saturating regime $n_H \geq 3$, for which the results become substantially more stable. This behavior is consistent with the saturation properties of the Hill function discussed above.

The normoxic case is shown in the left panel of Fig. S4. The largest changes occur for the lowest Hill coefficients. For $n_H = 1$, the frontier overlap with the $n_H = 4$ reference is 78%, the barycenter shifts by -0.083 Gy in dose and $+7.7$ days in interval, and the TTP deviation over the complete protocol domain is 0.694 years. For $n_H = 2$, the overlap increases to 88%, while the barycenter shifts are reduced to -0.050 Gy and $+4.1$ days and the RMSE decreases to 0.367 years. These deviations become substantially smaller once the Hill response enters the saturating regime. For $n_H = 3$, 5, and 6, the frontier overlaps are 95%, 97%, and 95%, respectively, with dose shifts

between -0.012 and $+0.006$ Gy and interval shifts between -2.0 and $+1.6$ days. The corresponding TTP RMSE values remain small, at 0.146, 0.098, and 0.168 years.

Importantly, the qualitative organization of the normoxic frontier is also preserved for $n_H \geq 3$. The near-optimal protocols remain broadly distributed in the dose–interval plane, and the reference standard-of-care schedule ($d = 1.8$ Gy, $\Delta = 1$ day) remains representative of the high-performing region. Thus, substantial deviations from the standard-of-care schedule do not provide a marked advantage under normoxic conditions. The persistence of this pattern across the sufficiently saturating Hill coefficients further supports the robustness of the normoxic treatment-selection result with respect to the choice of n_H.

The hypoxic case, shown in the right panel of Fig. S4, displays the same separation between low and sufficiently large Hill coefficients, although the effect of using a poorly saturating response is stronger. For $n_H = 1$, the overlap with the reference frontier falls to 15%, the barycenter shifts by $+0.910$ Gy and -6.7 days, and the TTP RMSE reaches 0.965 years. The $n_H = 2$ case is intermediate, with 57% overlap, a dose shift of $+0.287$ Gy, an interval shift of only -0.1 days, and an RMSE of 0.411 years. In contrast, the results become substantially more stable for $n_H \geq 3$, although the agreement with the reference case remains slightly weaker than under normoxic conditions: the overlaps are 88%, 92%, and 90% for $n_H = 3$, 5, and 6, respectively; the absolute dose shifts do not exceed 0.036 Gy, the interval shifts remain within 0.8 days, and the TTP RMSE values are 0.115, 0.074, and 0.105 years.

The hypoxic frontier also preserves the treatment-selection pattern identified in the main analysis. For $n_H \geq 3$, its geometry and characteristic location remain distinct from the normoxic case and retain the shift toward the metronomic protocol regime. Therefore, varying n_H within the saturating range does not remove the qualitative difference between normoxic and hypoxic protocol selection. In this sense, the effect associated with the oxygenation state remains clearly distinguishable from the comparatively small perturbations produced by changing the Hill coefficient within $n_H \geq 3$.

Overall, the sensitivity analysis shows a clear separation between the low-Hill cases and the effectively saturating regime. For $n_H = 3, 5, 6$, the therapeutic efficiency frontiers remain close to the $n_H = 4$ reference, their TTP-weighted barycenters undergo only small displacements, and the predicted TTP values remain quantitatively similar. Most importantly, the characteristic difference between the normoxic and hypoxic treatment-selection patterns is preserved, including the limited benefit of moving away from the standard-of-care schedule under normoxia and the preference for more metronomic protocols under hypoxia. The larger deviations observed for $n_H = 1$ and $n_H = 2$ are consistent with Hill responses that approach saturation too gradually to reproduce the intended oxygen-dependent behavior up to the normoxic level O_M. Since $n_H = 4$ was selected to provide an adequately saturating oxygen response over the relevant oxygen range, these results suggest that the main conclusions of the study are not strongly dependent on this specific choice.

Supporting figures

The following figures provide complementary material supporting the analyses presented in the main text and in the preceding sections of the Supporting Information.

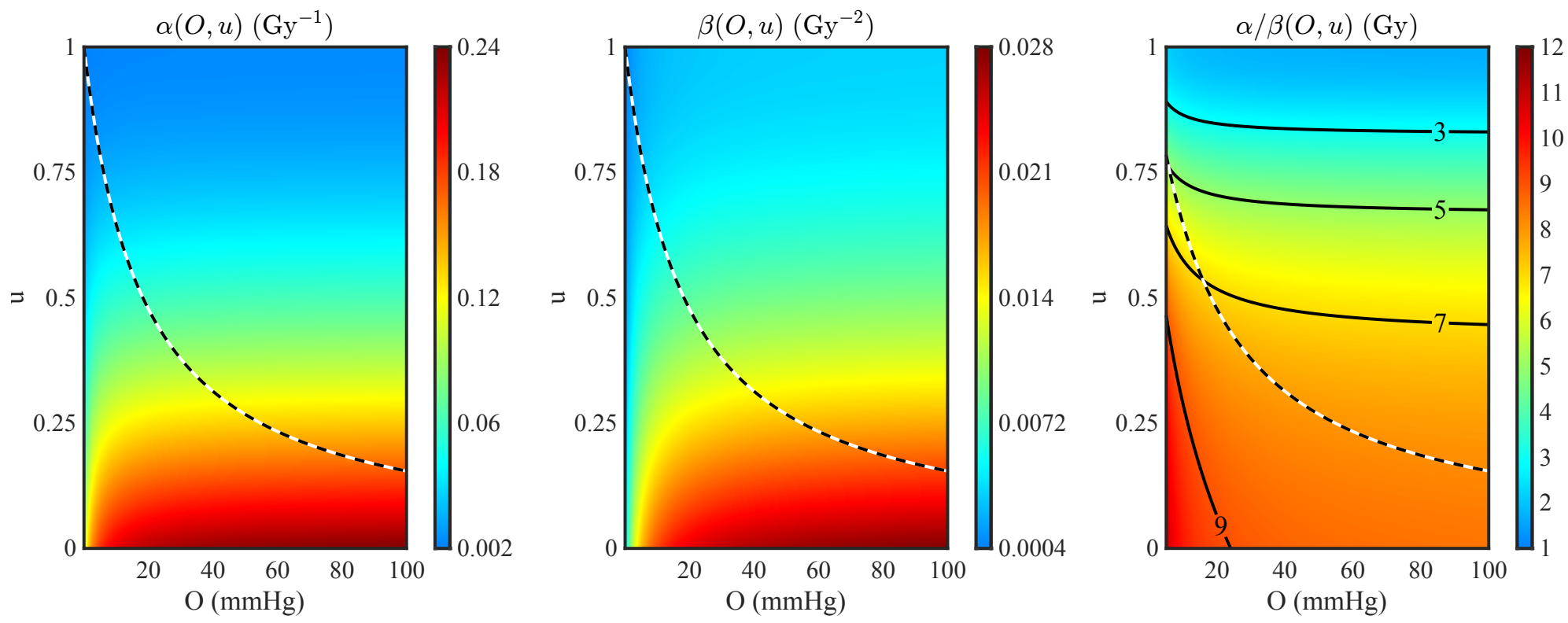

Fig. S1. Influence of oxygen concentration and phenotypic state on the radiosensitivity parameters of the modified LQ model defined in Eq. (6). **Left:** values of $\alpha(O, u)$. **Center:** values of $\beta(O, u)$. **Right:** corresponding α/β ratio, with contour lines highlighting constant-ratio levels. The dotted curve denotes the fittest phenotype $u = u^*$ (see Section S3) as a function of oxygen concentration. Although decreasing oxygen increases α/β at fixed phenotype, the ratio decreases along the u^* trajectory because hypoxia simultaneously selects for more resistant phenotypes.

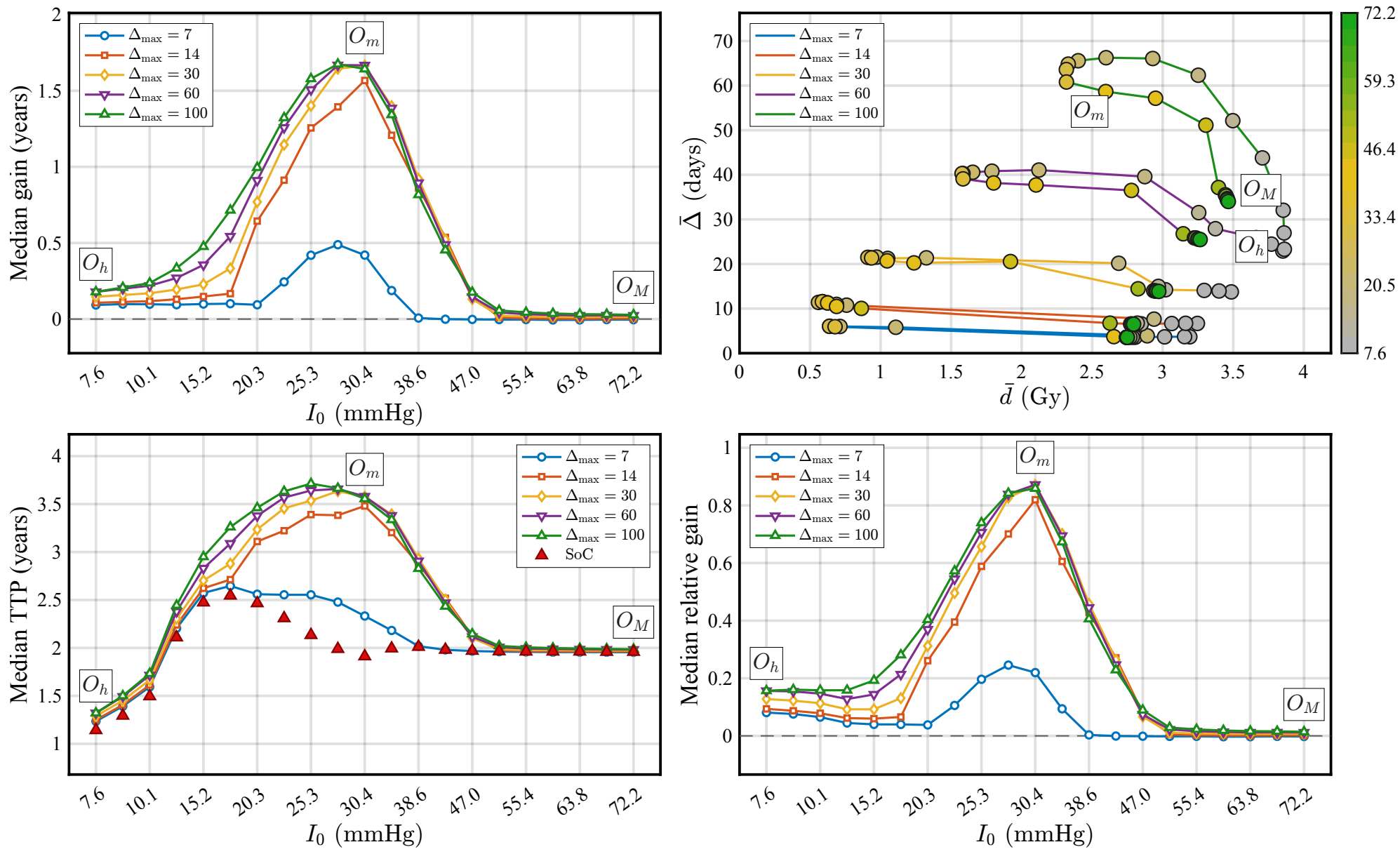

Fig. S2. Schedule-domain robustness under restricted inter-fraction intervals. Dose–interval scans were repeated under the same BED constraint for $\Delta_{\max} \in \{7, 14, 30, 60, 100\}$ days, with $\Delta_{\max} = 100$ corresponding to the extended range used in the main-text dose–interval maps. For each pair $(I_0, \Delta_{\max})$, the therapeutic efficiency frontier is recomputed after restricting the admissible inter-fraction interval range, and summary statistics are evaluated over the corresponding near-optimal protocols. **Top-left:** median TTP gain over SoC. **Top-right:** TTP-weighted frontier barycenters $(\bar{d}, \bar{\Delta})$ in the dose–interval plane; lines correspond to different values of $\Delta_{\max}$, while marker colors encode oxygenation levels, as indicated by the colorbar. **Bottom-left:** median TTP of near-optimal schedules, together with the SoC baseline. **Bottom-right:** median gain normalized by the corresponding SoC TTP baseline. Qualitative trends, including the intermediate-hypoxia window and barycenter shift, persist as $\Delta_{\max}$ decreases.

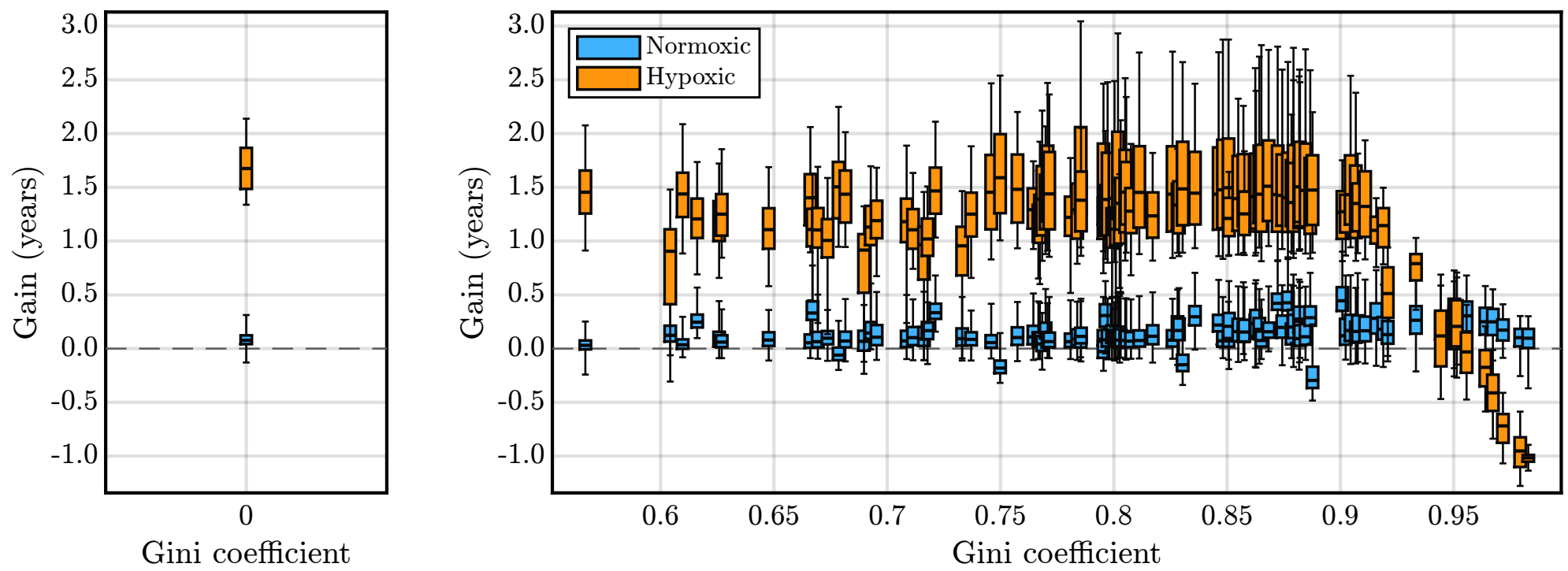

Fig. S3. Unbinned effect of spatial oxygen-source heterogeneity on protocols selected under uniform-supply assumptions. For each $I_0 \in \{O_M, O_m\}$, TTP gains are computed as in main-text Fig. 5 for protocols belonging to the uniform-source frontier $\mathcal{F}_u(I_0)$, and evaluated under either the corresponding uniform source or heterogeneous oxygen-source configurations, without re-optimization. Gains are measured relative to the standard-of-care protocol under the same oxygenation regime and spatial configuration. **Left:** uniform-source reference cases, for which the Gini coefficient is zero. **Right:** heterogeneous configurations shown individually, without pooling them into Gini bins. Each box summarizes the distribution of gains over the selected frontier protocols $\mathcal{P} \in \mathcal{F}_u(I_0)$ for a single oxygen-source realization $V^{(m)}_{O,I_0}(x)$, with spatial heterogeneity quantified by the corresponding Gini coefficient $\mathcal{G}^{(m)}_{I_0}$. Blue boxes indicate normoxic conditions ($I_0 = O_M$); orange boxes indicate moderately hypoxic conditions ($I_0 = O_m$). Boxes represent the interquartile range, central lines denote medians, and whiskers indicate minimum and maximum gains. The unbinned representation confirms the same regime-dependent trend observed in the binned analysis, showing that binning is used only to improve readability.

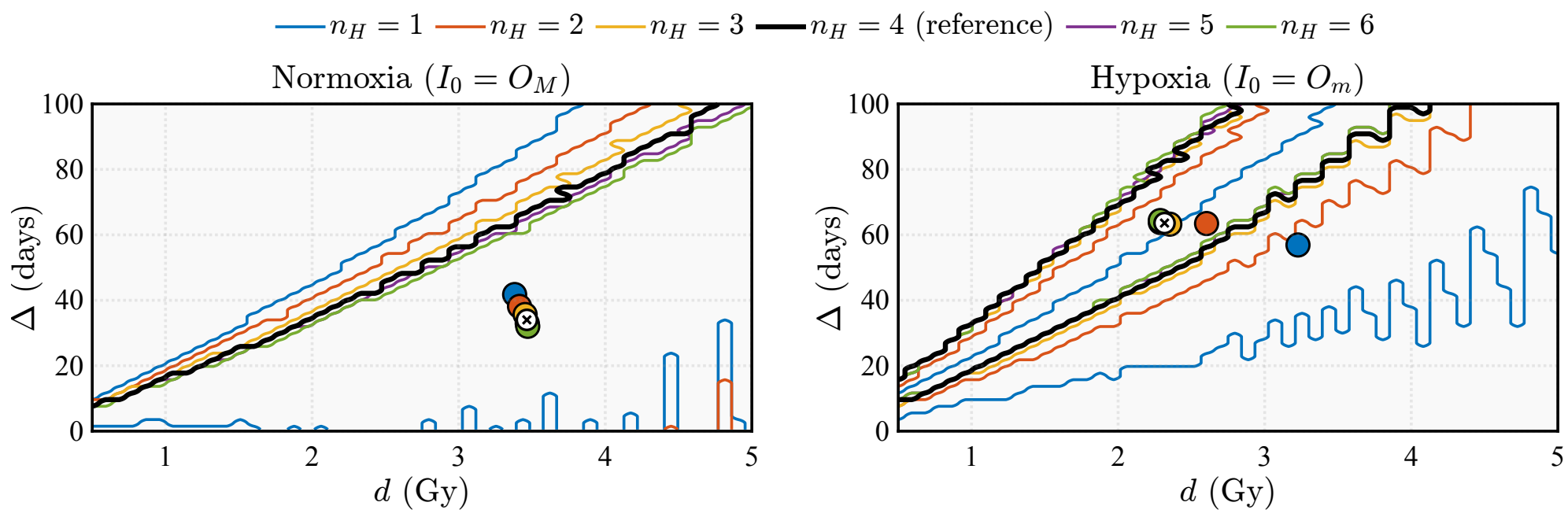

n_H	Overlap (%)	Dose shift (Gy)	Interval shift (days)	TTP deviation (years)
1	78	-0.083	+7.7	0.694
2	88	-0.050	+4.1	0.367
3	95	-0.012	+1.6	0.146
5	97	+0.003	-1.0	0.098
6	95	+0.006	-2.0	0.168

n_H	Overlap (%)	Dose shift (Gy)	Interval shift (days)	TTP deviation (years)
1	15	+0.910	-6.7	0.965
2	57	+0.287	-0.1	0.411
3	88	+0.036	-0.3	0.115
5	92	-0.029	+0.2	0.074
6	90	-0.029	+0.8	0.105

Fig. S4. Sensitivity of the dose–interval therapeutic efficiency frontier to the Hill coefficient n_H. Complete dose–interval scans were repeated for $n_H \in \{1, 2, 3, 4, 5, 6\}$, keeping all other model parameters and simulation settings unchanged. **Left:** normoxic condition $I_0 = O_M$. **Right:** hypoxic condition $I_0 = O_m$. For each n_H and oxygenation condition, the therapeutic efficiency frontier $\mathcal{F}_{n_H}$ identifies the near-optimal protocols attaining at least 80% of the corresponding maximum TTP; colored curves show its boundary. Filled circles indicate the TTP-weighted barycenters $(\bar{d}_{n_H}, \bar{\Delta}_{n_H})$, while the reference case $n_H = 4$ is highlighted by the thicker black contour and the white marker with a black cross. The tables quantify deviations from $n_H = 4$ through the percentage overlap between frontiers and the dose and interval shifts of their TTP-weighted barycenters, while TTP deviation is quantified by the RMSE (in years), computed protocol by protocol over the complete dose–interval domain. Overall, for the sufficiently saturating responses $n_H \geq 3$, the frontiers retain a high overlap with the reference case, their barycenters remain close, and TTP deviations remain limited under both normoxic and hypoxic conditions. The larger deviations observed for $n_H = 1$ and $n_H = 2$ are associated with Hill profiles that approach saturation more gradually over the relevant oxygen range. These results suggest that the main treatment-selection conclusions are not strongly dependent on the specific reference choice $n_H = 4$.

References

Brüningk, S.C., Peacock, J., Whelan, C.J., Brady-Nicholls, R., Yu, H.-H.M., Sahebjam, S., Enderling, H.: Intermittent radiotherapy as alternative treatment for recurrent high grade glioma: a modeling study based on longitudinal tumor measurements. Scientific Reports **11**, 20219 (2021) https://doi.org/10.1038/s41598-021-99507-2

Del Monte, U.: Does the cell number 10(9) still really fit one gram of tumor tissue? Cell Cycle **8**(3), 505–506 (2009) https://doi.org/10.4161/cc.8.3.7608

Erdi, Y.E.: Limits of tumor detectability in nuclear medicine and PET. Molecular Imaging and Radionuclide Therapy **21**(1), 23–28 (2012) https://doi.org/10.4274/Mirt.138

Fowler, J.F.: The linear-quadratic formula and progress in fractionated radiotherapy. British Journal of Radiology **62**(740), 679–694 (1989) https://doi.org/10.1259/0007-1285-62-740-679

Freedman, G.M., White, J.R., Arthur, D.W., Li, X.A., Vicini, F.A.: Accelerated fractionation with a concurrent boost for early stage breast cancer. Radiotherapy and Oncology **106**(1), 15–20 (2013) https://doi.org/10.1016/j.radonc.2012.12.001

Henares-Molina, A., Benzekry, S., Lara, P.C., García-Rojo, M., Pérez-García, V.M., Martínez-González, A.: Non-standard radiotherapy fractionations delay the time to malignant transformation of low-grade gliomas. PLOS ONE **12**(6), 0178552 (2017) https://doi.org/10.1371/journal.pone.0178552

Lorenzi, T., Painter, K.J., Villa, C.: Phenotype structuring in collective cell migration: a tutorial of mathematical models and methods. Journal of Mathematical Biology **90**, 61 (2025) https://doi.org/10.1007/s00285-025-02223-y

McMahon, S.J.: The linear quadratic model: usage, interpretation and challenges. Physics in Medicine and Biology **64**(1), 01–01 (2018) https://doi.org/10.1088/1361-6560/aaf26a

Mohamed, O.A.A., Tesen, H.S., Hany, M., Sherif, A., Abdelwahab, M.M., Elnaggar, M.H.: The role of hypoxia on prostate cancer progression and metastasis. Molecular Biology Reports **50**(4), 3873–3884 (2023) https://doi.org/10.1007/s11033-023-08251-5

Ritter, M., Forman, J., Kupelian, P., Lawton, C., Petereit, D.: Hypofractionation for prostate cancer. Cancer Journal **15**(1), 1–6 (2009) https://doi.org/10.1097/PPO.0b013e3181976614

Stea, B., Gordon, J.: Clinically relevant biomarkers in targeted radiotherapy. Clinical and Experimental Metastasis **29**, 853–860 (2012) https://doi.org/10.1007/s10585-012-9523-z

van Leeuwen, C.M., Oei, A.L., Crezee, J., Bel, A., Franken, N.A.P., Stalpers, L.J.A., Kok, H.P.: The alfa and beta of tumours: a review of parameters of the linear-quadratic model, derived from clinical radiotherapy studies. Radiation Oncology **13**(1), 96 (2018) https://doi.org/10.1186/s13014-018-1040-z

Wang, J.Z., Guerrero, M., Li, X.A.: How low is the alpha/beta ratio for prostate cancer? International Journal of Radiation Oncology, Biology, Physics **55**(1), 194–203 (2003) https://doi.org/10.1016/S0360-3016(02)03828-2

Wagner, B.A., Venkataraman, S., Buettner, G.R.: The rate of oxygen utilization by cells. Free Radical Biology and Medicine **51**(3), 700–712 (2011) https://doi.org/10.1016/j.freeradbiomed.2011.05.024
